# Platinum(IV)-Loaded Degraded Glycol Chitosan as Efficient Platinum(IV) Drug Delivery Platform

**DOI:** 10.3390/pharmaceutics15041050

**Published:** 2023-03-24

**Authors:** Yvonne Lerchbammer-Kreith, Nadine S. Sommerfeld, Klaudia Cseh, Xian Weng-Jiang, Uchechukwu Odunze, Andreas G. Schätzlein, Ijeoma F. Uchegbu, Mathea S. Galanski, Michael A. Jakupec, Bernhard K. Keppler

**Affiliations:** 1 Institute of Inorganic Chemistry, Faculty of Chemistry, University of Vienna, Waehringer Strasse 42, 1090 Vienna, Austria; 2 School of Pharmacy, University College London, Brunswick Square 29-39, London WC1N 1AX, UK; 3 Research Cluster “Translational Cancer Therapy Research”, University of Vienna, Waehringer Strasse 42, 1090 Vienna, Austria

**Keywords:** platinum(IV) complexes, glycol chitosan, anticancer, drug delivery

## Abstract

A new class of anticancer prodrugs was designed by combining the cytotoxicity of platinum(IV) complexes and the drug carrier properties of glycol chitosan polymers: Unsymmetrically carboxylated platinum(IV) analogues of cisplatin, carboplatin and oxaliplatin, namely (OC-6-44)-acetatodiammine(3-carboxypropanoato)dichloridoplatinum(IV), (OC-6-44)-acetaodiammine(3-carboxypropanoato)(cyclobutane-1,1-dicarboxylato)platinum(IV) and (OC-6-44)-acetato(3-carboxypropanoato)(1R,2R-cyclohexane-1,2-diamine)oxalatoplatinum(IV) were synthesised and conjugated via amide bonding to degraded glycol chitosan (dGC) polymers with different chain lengths (5, 10, 18 kDa). The 15 conjugates were investigated with ^1^H and ^195^Pt NMR spectroscopy, and average amounts of platinum(IV) units per dGC polymer molecule with ICP-MS, revealing a range of 1.3–22.8 platinum(IV) units per dGC molecule. Cytotoxicity was tested with MTT assays in the cancer cell lines A549, CH1/PA-1, SW480 (human) and 4T1 (murine). IC_50_ values in the low micromolar to nanomolar range were obtained, and higher antiproliferative activity (up to 72 times) was detected with dGC-platinum(IV) conjugates in comparison to platinum(IV) counterparts. The highest cytotoxicity (IC_50_ of 0.036 ± 0.005 µM) was determined in CH1/PA-1 ovarian teratocarcinoma cells with a cisplatin(IV)–dGC conjugate, which is hence 33 times more potent than the corresponding platinum(IV) complex and twice more potent than cisplatin. Biodistribution studies of an oxaliplatin(IV)–dGC conjugate in non-tumour-bearing Balb/C mice showed an increased accumulation in the lung compared to the unloaded oxaliplatin(IV) analogue, arguing for further activity studies.

## 1. Introduction

Platinum(II)-based chemotherapy is indispensable in modern clinical oncology, with an expectedly growing global market for the three major drugs, cisplatin, carboplatin and oxaliplatin [1]. Despite the tremendous success of those three platinum(II) complexes, which are in global clinical use and together are integrated in about 50% of all chemotherapies [2], no other platinum complex has reached worldwide marketing approval. The major limitation of platinum(II)-based anticancer treatment is the minor selectivity towards tumour tissue, leading to severe side effects, especially nephrotoxicity and neurotoxicity [3,4]. The clinical efficacy is highly diminished by cancer resurgence and intrinsic as well as acquired therapy resistance [5]. The platinum(IV) prodrug strategy is one attempt to overcome the downsides of platinum(II) drugs. This strategy is driven by the possibility to overcome resistances and to reduce toxicities by introduction of two axial ligands, leading to higher kinetic inertness and less indiscriminate reactions with biological nucleophiles [6]. Additionally, platinum(IV) complexes follow the “activation by reduction” concept and evolve their cytotoxic properties by releasing their axial ligands [7]. The required reduction is especially facilitated by the characteristic acidic and oxygen-deficient environment in tumour tissue [8]. Initial investigation of mainly axially symmetric platinum(IV) compounds such as tetraplatin, iproplatin, LA-12 and, in particular, satraplatin, in clinical trials has remained unsuccessful [2,9]. Our group made significant progress in the diversification of the platinum(IV) ligand sphere over the last decade(s), with an exceptional focus on mixed axial ligands [10,11,12] and multifunctionality [13]. The platinum(IV) paradigm shifted towards the integration of moieties with drug-targeting abilities, easily accessible via the additional ligands, for optimising pharmacokinetic and -dynamic properties [14].

**Scheme 1:**
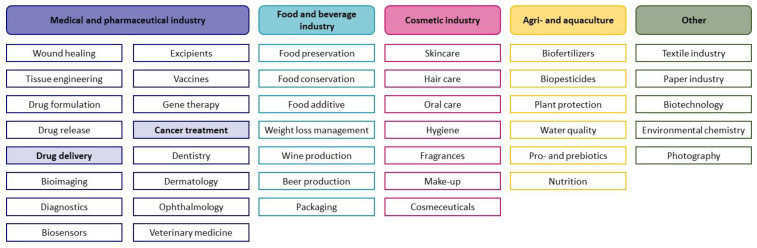
Overview of different application areas of chitosan and its derivatives (not exhaustive) [22].

The natural chitosan polymers as emerging drug-targeting moieties seem to be promising candidates for improved application and biodistribution of platinum(IV) complexes. They are extensively studied based on their advantageous properties, such as biodegradability, biocompatibility and their polyelectrolyte character [15,16]. The polysaccharide chitosan can be obtained from the natural source chitin, occurring in shells of crustaceans, exoskeletons of insects or cell walls of fungi, via deacetylation. However, harsh conditions from using hydroxides in combination with high temperatures during the deacetylation process can lead to a decrease in molecular weight. According to the desired final properties, chitosan with different degrees of acetylation (DA) can be obtained. Consequently, chitosan consists of two different monosaccharide units: N-acetyl-D-glucosamine and D-glucosamine, which are connected via 𝛽 (1→4) bonds [17,18]. The DA strongly influences the solubility. Whereas chitin with a very high DA lacks in solubility, chitosan with DA lower than 50 mol% is soluble in water under acidic conditions [19]. The solubility can be further improved by derivatisation, leading to high solubility in water at acidic and neutral pH and organic solvents, for derivatives such as glycol chitosan [17,20]. In addition to glycol chitosan, various derivatives such as different quaternised and alkylated chitosan polymers were synthesised and investigated based on the ease of modification of the chitosan structure via chemical and enzymatic procedures [17,21]. Due to the possibility of crosslinks with various reagents and the polycationic nature, chitosan can be obtained in various forms such as powders, tablets, solutions, gels, membranes or sponges, enabling numerous administration routes such as oral, parenteral and transdermal. Furthermore, chitosan’s antioxidant, anti-inflammatory, antibacterial, antimicrobial and antitumour properties in combination with its nontoxicity and biocompatibility are used in different application fields, reaching from food preservation to cosmetics (Scheme 1) [19,21,22]. Especially, medical purposes, such as chitosan being used as artificial skin and as a drug carrier, attract more and more attention [23]. Due to their nontoxicity, adsorption capacities and facile derivatisation [24], chitosan polymers are promising platforms for encapsulation or conjugation with different agents such as anticancer compounds. As a result of their nanometre size, the enhanced permeability and retention effect (EPR effect) can be exploited for passive tumour targeting. Due to defective angiogenesis during tumour growth, large gaps (600–800 nm) between endothelial cells of blood vessels are formed, and small polymers can enter tumour cells by endocytosis. Continuous accumulation of polymers is further supported by malfunctioning lymph drainage. Additionally, drug release is facilitated by the acidic and hypoxic environment in tumour tissue [25,26,27]. 

Accordingly, the combination of the drug delivery properties of chitosan polymers and platinum-based anticancer agents opens up a promising strategy for reducing systemic toxicity and overcoming resistance. Recently, platinum(IV) analogues of cisplatin encapsulated in modified chitosan nanoparticles showed higher potency in cisplatin-sensitive as well as resistant lung cancer cells compared to cisplatin alone [28]. Furthermore, hydrophobically modified glycol chitosan nanocarriers loaded with cisplatin were investigated recently in in vivo studies with tumour-bearing mice. The accumulation in cancerous tissue was demonstrated with near-infrared fluorescence imaging technology, and higher tumour-inhibiting activity combined with reduced adverse effects was found in comparison to free cisplatin [29]. Reduced adverse effects compared to cisplatin were also detected in in vivo studies by using self-assembled chitosan polymers loaded with cisplatin. Renal, hepatic and testicular cells were monitored by physiological and histopathological investigations after the application of cisplatin-loaded chitosan polymers, revealing improved conditions of the respective cells compared to the cisplatin control. Additionally, the treatment with cisplatin increased the serum levels of different liver, kidney and testicular markers, whereas the application of cisplatin–chitosan nanoparticles revealed normal levels [30]. Finally, self-assembled micelles of N-octyl-N,O-succinyl chitosan loaded with cisplatin significantly decreased cisplatin-induced apoptosis in renal cells and maintained cell viability. In addition to the reduced nephrotoxicity compared to cisplatin, the platinum(II)-loaded chitosan derivatives showed moderate anticancer activity [31]. 

**Scheme 2:**
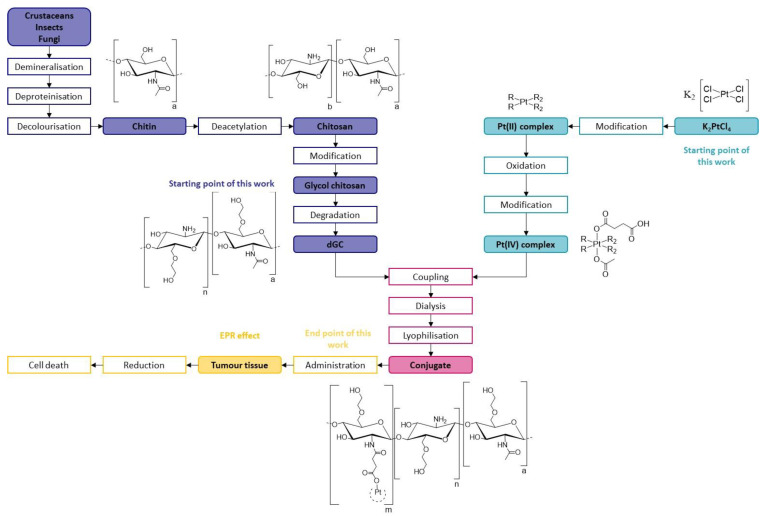
Overview of process steps and delivery concept of platinum(IV)–dGC conjugates [26,32]. In the presented work, the synthesis of dGC polymers started with degradation of glycol chitosan, whereas platinum(IV) complexes were obtained by modification and oxidation of K_2_PtCl_4_. Additionally, in vivo studies were performed in non-tumour-bearing mice.

Driven by these promising results in combination with the favourable solubility profile as well as the possibility of surface modifications and variation of molecular weights, we chose glycol chitosan as drug delivery system. We aimed to synthesise, characterise and investigate platinum(IV)-loaded degraded glycol chitosan (dGC) nanoparticles. In contrast to previously published studies using encapsulated cisplatin, platinum(IV) analogues of cisplatin, carboplatin and oxaliplatin were conjugated via amide bonds to the surface of dGC polymers with three different chain lengths. The various conjugates were characterised with NMR spectroscopy and inductively coupled plasma MS (ICP-MS). The conjugates were investigated for their cytotoxicity in different human and murine cancer cell lines, leading to a comparative in vivo study of the most promising oxaliplatin-based conjugate in comparison to its platinum(IV) analogue (Scheme 2).

## 2. Materials and Methods

### 2.1. Materials

All solvents and chemicals were purchased from commercial suppliers and were used as received. The following chemicals were used without further purification for the synthesis: K_2_[PtCl_4_] (Assay: 46.69% Pt) (Johnson Matthey, Zurich, Switzerland), glycol chitosan (Assay: 78.2%) (Wako, Osaka, Japan), succinic anhydride (≥99%) (Sigma Aldrich, Steinheim, Germany), acetic anhydride (≥97.0%) (Fisher Scientific, Schwerte, Germany), absolute DMF (99.8%, extra dry, water <50 ppm) (Acros Organics, Geel, Belgium), absolute DMSO (99.7%, extra dry, water <50 ppm) (Acros Organics, Geel, Belgium), triethylamine (99%) (Acros Organics, Geel, Belgium). 

Trial Kit Spectra/Por^®^ 3 (molecular weight cut off (MWCO) = 3.5 kDa) and Spectra/Por^®^ 7 (MWCO = 1.0 kDa) dialysis tubing was purchased from Carl Roth (Karlsruhe, Germany). All aqueous solutions were performed with Milli-Q water (18.2 MΩ cm, Milli-Q Advantage). Reactions containing platinum complexes were conducted with glass-coated magnetic stirring bars and protected from light.

### 2.2. NMR Spectroscopy

NMR spectra were measured with a Bruker Avance NEO 500 MHz NMR spectrometer at 500.32 (^1^H), 125.81 (^13^C), 50.70 (^15^N) and 107.55 MHz (^195^Pt) in d_6_-DMSO, d_7_-DMF or D_2_O at 298 K. ^1^H and ^13^C NMR spectra were measured relative to the solvent resonances (d_6_-DMSO: δ = 2.50 ppm (^1^H), δ = 39.51 ppm (^13^C); d_7_-DMF: δ = 2.75 ppm (high field signal) (^1^H), δ = 29.76 ppm (high field signal) (^13^C); D_2_O: δ = 4.79 ppm (^1^H)). K_2_[PtCl_4_] and NH_4_Cl were used as external standards for ^195^Pt und ^15^N NMR spectra. 

### 2.3. RP-HPLC

An Agilent 1200 Series system equipped with a XBridge^®^ Prep C18 10 µm OBD^TM^ Column (19 mm × 250 mm) and an Atlantis^®^ T3 Prep 10 µm OBD^TM^ Column (19 mm × 250 mm) from Waters was used for preparative RP-HPLC. All runs were performed with a mixture of Milli-Q water and acetonitrile, or Milli-Q water and methanol with addition of 0.1% formic acid.

### 2.4. Elemental Analysis

The elemental analysis (C, H, N) of the platinum(IV) complexes was performed by the Microanalytical Laboratory of the Faculty of Chemistry of the University of Vienna with an Eurovector EA3000 elemental analyser. The obtained and calculated values are within a ±0.4% range. 

### 2.5. Synthesis

#### 2.5.1. Platinum(IV) Complexes

The dihydroxidoplatinum(IV) analogues of cisplatin, carboplatin and oxaliplatin were synthesised according to standard procedures [33,34,35]. The carboxylation of the platinum(IV) compounds was adapted from previously published synthetic pathways [13,36]. 

##### General Procedure 1: Carboxylation of Dihydroxidoplatinum(IV) Complexes with Succinic Anhydride 

The corresponding dihydroxidoplatinum(IV) complex and succinic anhydride were stirred overnight in absolute DMSO under an argon atmosphere at 50 °C. The solvent was removed under reduced pressure.

1. (OC-6-44)-Diammine(3-carboxypropanoato)dichloridohydroxidoplatinum(IV) (1)

The reaction was performed according to general procedure 1, using diamminedichloridodihydroxidoplatinum(IV) (655 mg, 1.96 mmol, 1 eq), succinic anhydride (196 mg, 1.96 mmol, 1 eq) and absolute DMSO (10 mL). Yield: 845 mg (crude product used for next step).

2. (OC-6-44)-Diammine(3-carboxypropanoato)(cyclobutane-1,1-dicarboxylato)hydroxidoplatinum(IV) (3)

The reaction was performed according to general procedure 1 using diammine(cyclobutane-1,1-dicarboxylato)dihydroxidoplatinum(IV) (1.416 g, 3.49 mmol, 1 eq), succinic anhydride (349.9 mg, 3.49 mmol, 1 eq) and absolute DMSO (80 mL). Yield: 1.564 g (crude product used for next step).

3. (OC-6-44)-(3-carboxypropanoato)(1R,2R-cyclohexane-1,2-diamine)hydroxidooxalatoplatinum(IV) (5)

The reaction was performed according to general procedure 1 using (1R,2R-cyclohexane-1,2-diamine)dihydroxidooxalatoplatinum(IV) (337 mg, 0.78 mmol, 1 eq.), succinic anhydride (79 mg, 0.78 mmol, 1 eq) and absolute DMSO (50 mL). The crude product was purified by using preparative RP-HPLC (column: XBridge^®^, isocratic, MeOH:Milli-Q water = 5:95 + 0.1% formic acid). Yield: 217 mg (52%). ^1^H NMR (d_6_-DMSO): δ = 12.08 (b, 2H, O*H*), 8.43 (b, 1H, N*H*_2_), 8.10 (b, 1H, N*H*_2_), 7.82 (b, 1H, N*H*_2_), 7.08 (b, 1H, N*H*_2_); overlapping of the C*H* (DACH) signal with the DMSO solvent peak, 2.40–2.44 (m, 2H, C*H*_2_, succinato), 2.34–2.39 (m, 2H, C*H*_2_, succinato), 2.00–2.10 (m, 2H, C*H*_2_, DACH), 1.41–1.53 (m, 3H, C*H*_2_, DACH), 1.25–1.36 (m, 1H, C*H*_2_, DACH), 1.08–1.15 (m, 2H, C*H*_2_, DACH) ppm.

##### General Procedure 2: Introduction of the Acetato Ligand

The (3-carboxypropanoato)hydroxidoplatinum(IV) complex was suspended in absolute DMF. Acetic anhydride was added, and the reaction mixture was stirred overnight at 45 °C under an argon atmosphere. The solvent was removed, and the crude product was purified via preparative RP-HPLC. The final product was freeze-dried.

1. (OC-6-44)-Acetatodiammine(3-carboxypropanoato)dichloridoplatinum(IV) (2)

The reaction was performed according to general procedure 2 using **1** (845 mg, impure, 1.95 mmol, 1eq), acetic anhydride (1 mL, 10.6 mmol, 5.4 eq) and absolute DMF (15 mL). Preparative RP-HPLC (column: Atlantis^®^, isocratic, MeOH:Milli-Q water = 1:95 + 0.1% formic acid). Yield: 157 mg. ^1^H NMR (d_6_-DMSO): δ = 6.50 (b, 6H, N*H*_3_); one of the C*H*_2_ signals of the succinato ligand in part overlapped with the DMSO solvent peak, 2.32–2.39 (m, 2H, C*H*_2_, succinato), 1.90 (s, 3H, C*H*_3_) ppm. ^13^C NMR (d_6_-DMSO): δ = 179.6 (*C*=O, succinato), 178.2 (*C*=O, acetato), 173.8 (*C*=O, succinato), 30.5 (*C*H_2_, succinato), 29.8 (*C*H_2_, succinato), 22.8 (*C*H_3_) ppm. ^15^N NMR (d_6_-DMSO): δ = −41.2 (*N*H_3_) ppm. ^195^Pt NMR (d_6_-DMSO): δ = 2857 ppm. Elemental analysis: C_6_H_14_Cl_2_N_2_O_6_Pt∙H_2_O; calcd. C 14.58, H 3.26, N 5.67, found C 14.40, H 3.14, N 5.64.

2. (OC-6-44)-Acetaodiammine(3-carboxypropanoato)(cyclobutane-1,1-dicarboxylato)platinum(IV) (4)

The reaction was performed according to general procedure 2 using **3** (200 mg, 0.40 mmol, 1 eq), acetic anhydride (0.5 mL, 0.53 mmol, 1.3 eq) and absolute DMF (10 mL). Preparative RP-HPLC (column: XBridge^®^, isocratic, MeOH:Milli-Q water = 17:83 + 0.1% formic acid). Yield: 107 mg. ^1^H NMR (d_7_-DMF): δ = 6.76 (t, ^1^J (^14^N, ^1^H) = 52.8 Hz, 6H, N*H*_3_), 2.62 (m, 4H, C*H*_2_-C, cyclobutyl), 2.51 (m, 2H, C*H*_2_, succinato), 2.45 (m, 2H, C*H*_2_, succinato), 1.89 (p, ^3^J (^1^H, ^1^H = 8.0 Hz), 2H, C*H*_2_, cyclobutyl), 1.86 (s, 3H, C*H*_3_) ppm. ^13^C NMR (d_7_-DMF): δ = 179.8 (*C*=O, succinato), 178.3 (*C*=O, acetato), 177.0 (*C*=O, dicarboxylato), 174.1 (*C*=O, succinato), 56.5 (*C*, cyclobutyl), 32.4 (*C*H_2_-C, cyclobutyl), 31.8 (*C*H_2_-C, cyclobutyl), 30.7 (*C*H_2_, succinato); one of the C*H*_2_ signals of the succinato ligand overlapped with the DMF solvent peak, 22.0 (*C*H_3_), 16.2 (*C*H_2_, cyclobutyl) ppm. ^15^N NMR (d_7_-DMF): δ = −54.3 (*N*H_3_) ppm. ^195^Pt NMR (d_7_-DMF): δ = 3562 ppm. Elemental analysis: C_12_H_20_N_2_O_10_Pt; calcd. C 26.33, H 3.68, N 5.12, found C 25.99, H 3.46, N 5.00.

3. (OC-6-44)-Acetato(3-carboxypropanoato)(1R,2R-cyclohexane-1,2-diamine)oxalatoplatinum(IV) (6)

The reaction was performed according to general procedure 2 using **5** (217 mg, 0.41 mmol, 1 eq), acetic anhydride (0.5 mL, 0.53 mmol, 1.3 eq) and absolute DMF (10 mL). Preparative RP-HPLC (column: XBridge^®^, isocratic, ACN:Milli-Q water = 5:95 + 0.1% formic acid). Yield: 74 mg (32%). ^1^H NMR (d_6_-DMSO): δ = 12.11 (b, 1H, O*H*), 8.29 (m, 4H, N*H*_2_), 2.55–2.63 (m, 2H, C*H*, DACH); one of the C*H*_2_ signals of the succinato ligand overlapped with the DMSO solvent peak, 2.38 (m, 2H, C*H*_2_, succinato), 2.07–2.15 (m, 2H, C*H*_2_, DACH), 1.96 (s, 3H, C*H*_3_), 1.33–1.56 (m, 4H, C*H*_2_, DACH), 1.10–1.22 (m, 2H, C*H*_2_, DACH) ppm. ^13^C NMR (d_6_-DMSO): δ = 179.6 (*C*=O, succinato), 178.6 (*C*=O, acetato), 173.7 (*C*=O, succinato), 163.41 (*C*=O, oxalato), 163.35 (*C*=O, oxalato), 60.9 (*C*H, DACH), 60.8 (*C*H, DACH), 30.9 (*C*H_2_, DACH), 30.8 (*C*H_2_, DACH), 30.5 (*C*H_2_, succinato), 29.6 (*C*H_2_, succinato), 23.5 (*C*H_2_, DACH), 23.4 (*C*H_2_, DACH), 23.0 (*C*H_3_) ppm. ^15^N NMR (d_6_-DMSO): δ = −6.4 (*N*H_2_) ppm. ^195^Pt NMR (d_6_-DMSO): δ = 3242 ppm. Elemental analysis: C_14_H_22_N_2_O_10_Pt∙H_2_O; calcd. C 28.43, H 4.09, N 4.74, found C 28.46, H 3.82, N 4.92.

#### 2.5.2. Degradation of Glycol Chitosan (dGC)

The degradation of glycol chitosan (GC) was carried out as previously reported [37]. GC polymer chains (MW~120 kDa, 20 g) were suspended in HCl (4 M, 760 mL) and stirred for 20 min at room temperature. Subsequently, the solution was degraded with continuous stirring by means of a water bath at 50 °C. In order to obtain specific molecular weights, the following three time points were chosen: 2 h (for MW~18 kDa), 8 h (for MW~10 kDa), and 24 h (for MW~5 kDa). The solution was allowed to cool down (30 min) and dialysed exhaustively against water using a dialysis membrane (MWCO = 3.5 kDa). The dialysed solution was then freeze-dried to yield a white fibrous solid. Yield = 45–80%.

#### 2.5.3. Platinum(IV)–dGC Conjugates

##### General Procedure 3: Conjugation of Platinum(IV) Complex to dGC Polymer 

The corresponding platinum(IV) complex was dissolved in DMSO (1 mL). CDI was added, and the mixture was stirred for about 15 min. The dGC polymer was dissolved in DMSO (1 mL) and trimethylamine (TEA). Both solutions were merged together and stirred overnight at room temperature. Two slightly different purification steps were performed:

(a) Dialysis was conducted against distilled water and 0.1 M hydrochloric acid (in each case 6 changes within 24 h). Dialysis tubing with MWCO = 1.0 kDa for **P1** and MWCO = 3.5 kDa for **P2** and **P3** was used. The product was obtained via lyophilisation and stored under an argon atmosphere at −30 °C.
(b) Dialysis was conducted against Milli-Q water (10 changes within 12 h) with dialysis tubing with MWCO = 1.0 kDa for **P1** and MWCO = 3.5 kDa for **P2** and **P3**. Afterwards the solution was adjusted to pH = 3 with hydrochloric acid and freeze-dried. The product was stored under an argon atmosphere at −30 °C.

1. Complex 2 Coupled to dGC P1 (C1)

The reaction was performed according to general procedure 3b using **2** (35 mg, 0.70 mmol, 1 eq), CDI (60 mg, 0.37 mmol, 5 eq), dGC **P1** (60 mg, 0.29 mmol, 4 eq/monomer unit) and trimethylamine (41 µL, 0.29 mmol, 4 eq). Yield: 46 mg. ICP-MS (Pt): 77.7 g/kg. ^1^H NMR (D_2_O): δ = 4.86 (b, O-C*H*-O, backbone of dGC), 3.46–4.28 (m, backbone of dGC), 3.19 (b, C*H*-NH/NH_2_, backbone of dGC), 2.56–2.75 (m, C*H*_2_, succinato), 2.12 (s, C*H*_3_), 2.05–2.11 (m, residues of acetylated dGC monomers) ppm. ^195^Pt NMR (D_2_O): δ = 2705 ppm.

2. Complex 2 Coupled to dGC P2 (C2)

The reaction was performed according to general procedure 3a using **2** (73 mg, 0.15 mmol, 1 eq), CDI (125 mg, 0.77 mmol, 5 eq), dGC **P2** (60 mg, 0.29 mmol, 2 eq/monomer unit) and trimethylamine (76 µL, 0.55 mmol, 4 eq). Yield: 75 mg. ICP-MS (Pt): 160.4 g/kg. ^1^H NMR (D_2_O): δ = 4.87 (b, O-C*H*-O, backbone of dGC), 3.56–4.10 (m, backbone of dGC), 3.19 (b, C*H*-NH/NH_2_, backbone of dGC), 2.56–2.70 (m, C*H*_2_, succinato), 2.11 (s, C*H*_3_), 2.04–2.10 (m, residues of acetylated dGC monomers) ppm. ^195^Pt NMR (D_2_O): δ = 2705 ppm.

3. Complex 4 Coupled to dGC P1 (C3)

The reaction was performed according to general procedure 3b using **4** (70 mg, 0.13 mmol, 1 eq), CDI (103 mg, 0.64 mmol, 5 eq), dGC **P1** (52 mg, 0.26 mmol, 2 eq/monomer unit) and trimethylamine (71 µL, 0.51 mmol, 4 eq). Yield: 97 mg. ICP-MS (Pt): 42.6 g/kg. ^1^H NMR (D_2_O): δ = 4.87 (b, O-C*H*-O, backbone of dGC), 3.46–4.14 (m, backbone of dGC), 3.19 (b, C*H*-NH/NH_2_, backbone of dGC), 2.64–2.71 (m, C*H*_2_, succinato + C*H*_2_-C, cyclobutyl), 2.58–2.62 (m, C*H*_2_, succinato), 2.08 (s, C*H*_3_), 2.05–2.11 (m, residues of acetylated dGC monomers), 2.03 (m, C*H*_2_, cyclobutyl) ppm. ^195^Pt NMR (D_2_O): δ = 3508 ppm.

4. Complex 4 Coupled to dGC P1 (C4)

The reaction was performed according to general procedure 3b using **4** (35 mg, 0.6 mmol, 1 eq), CDI (52 mg, 0.32 mmol, 5 eq), dGC **P1** (52 mg, 0.26 mmol, 4 eq/monomer unit) and trimethylamine (35 µL, 0.26 mmol, 4 eq). Yield: 51 mg. ICP-MS (Pt): 84.5 g/kg. ^1^H NMR (D_2_O): δ = 4.87 (b, O-C*H*-O, backbone of dGC), 3.57–4.11 (m, backbone of dGC), 3.19 (b, C*H*-NH/NH_2_, backbone of dGC), 2.64–2.71 (m, C*H*_2_, succinato + C*H*_2_-C, cyclobutyl), 2.57–2.62 (m, C*H*_2_, succinato), 2.08 (s, C*H*_3_), 2.05–2.11 (m, residues of acetylated dGC monomers), 2.03 (m, C*H*_2_, cyclobutyl) ppm. ^195^Pt NMR (D_2_O): δ = 3508 ppm.

5. Complex 4 Coupled to dGC P2 (C5)

The reaction was performed according to general procedure 3b using **4** (70 mg, 0.13 mmol, 1 eq), CDI (104 mg, 0.64 mmol, 5 eq), dGC **P2** (53 mg, 0.26 mmol, 2 eq/monomer unit) and trimethylamine (71 µL, 0.51 mmol, 4 eq). Yield: 88 mg. ICP-MS (Pt): 171.5 g/kg. ^1^H NMR (D_2_O): δ = 4.87 (b, O-C*H*-O, backbone of dGC), 3.47–4.19 (m, backbone of dGC), 3.18 (b, C*H*-NH/NH_2_, backbone of dGC), 2.64–2.72 (m, C*H*_2_, succinato + C*H*_2_-C, cyclobutyl), 2.58–2.62 (m, C*H*_2_, succinato), 2.08 (s, C*H*_3_), 2.05–2.11 (m, residues of acetylated dGC monomers), 2.03 (m, C*H*_2_, cyclobutyl) ppm. ^195^Pt NMR (D_2_O): δ = 3509 ppm.

6. Complex 6 Coupled to dGC P1 (C6)

The reaction was performed according to general procedure 3b using **6** (70 mg, 0.12 mmol, 1 eq), CDI (99 mg, 0.61 mmol, 5 eq), dGC **P1** (50 mg, 0.24 mmol, 2 eq/monomer unit) and TEA (68 µL, 0.49 mmol, 4 eq). Yield: 83 mg. ICP-MS (Pt): 100.3 g/kg. ^1^H NMR (D_2_O): δ = 4.87 (b, O-C*H*-O, backbone of dGC), 3.59–4.14 (m, backbone of dGC), 3.18 (b, C*H*-NH/NH_2_, backbone of dGC), 2.84–2.95 (m, C*H*, DACH), 2.64–2.69 (m, C*H*_2_, succinato), 2.59–2.64 (m, C*H*_2_, succinato), 2.27–2.34 (m, C*H*_2_, DACH), 2.08 (s, C*H*_3_), 2.06–2.08 (m, residues of acetylated dGC monomers, overlapped with acetato signal), 1.53–1.70 (m, C*H*_2_, DACH), 1.20–1.32 (m, C*H*_2_, DACH) ppm. ^195^Pt NMR (D_2_O): δ = 3213 ppm.

7. Complex 6 Coupled to dGC P1 (C7)

The reaction was performed according to general procedure 3b using **6** (70 mg, 0.12 mmol, 1 eq), CDI (99 mg, 0.61 mmol, 5 eq), dGC **P1** (50 mg, 0.24 mmol, 2 eq/monomer unit) and TEA (68 µL, 0.49 mmol, 4 eq). Yield: 71 mg. ICP-MS (Pt): 148.1 g/kg. ^1^H NMR (D_2_O): δ = 4.87 (b, O-C*H*-O, backbone of dGC), 3.60–4.08 (m, backbone of dGC), 3.18 (b, C*H*-NH/NH_2_, backbone of dGC), 2.83–2.96 (m, C*H*, DACH), 2.64–2.70 (m, C*H*_2_, succinato), 2.59–2.63 (m, C*H*_2_, succinato), 2.26–2.34 (m, C*H*_2_, DACH), 2.07 (s, C*H*_3_), 2.05–2.08 (m, residues of acetylated dGC monomers), 1.53–1.71 (m, C*H*_2_, DACH), 1.23–1.32 (m, C*H*_2_, DACH) ppm. ^195^Pt NMR (D_2_O): δ = 3214 ppm.

8. Complex 6 Coupled to dGC P2 (C8)

The reaction was performed according to general procedure 3a using **6** (70 mg, 0.12 mmol, 1 eq), CDI (104 mg, 0.63 mmol, 5 eq), dGC **P2** (50 mg, 0.24 mmol, 2 eq/monomer unit) and TEA (64 µL, 0.46 mmol, 4 eq). Yield: 58 mg. ICP-MS (Pt): 73.4 g/kg. ^1^H NMR (D_2_O): δ = 4.87 (b, O-C*H*-O, backbone of dGC), 3.55–4.30 (m, backbone of dGC), 3.19 (b, C*H*-NH/NH_2_, backbone of dGC), 2.83–2.98 (m, C*H*, DACH), 2.64–2.74 (m, C*H*_2_, succinato), 2.59–2.64 (m, C*H*_2_, succinato), 2.26–2.36 (m, C*H*_2_, DACH), 2.08 (s, C*H*_3_), 2.04–2.11 (m, residues of acetylated dGC monomers), 1.53–1.71 (m, C*H*_2_, DACH), 1.20–1.32 (m, C*H*_2_, DACH) ppm. ^195^Pt NMR (D_2_O): δ = 3213 ppm.

9. Complex 6 Coupled to dGC P2 (C9)

The reaction was performed according to general procedure 3b using **6** (70 mg, 0.12 mmol, 1 eq), CDI (99 mg, 0.61 mmol, 5 eq), **P2** (50 mg, 0.24 mmol, 2 eq/monomer unit) and TEA (68 µL, 0.49 mmol, 4 eq). Yield: 96 mg. ICP-MS (Pt): 116.5 g/kg. ^1^H NMR (D_2_O): δ = 4.87 (b, O-C*H*-O, backbone of dGC), 3.56–4.15 (m, backbone of dGC), 3.19 (b, C*H*-NH/NH_2_, backbone of dGC), 2.83–2.95 (m, C*H*, DACH), 2.65–2.70 (m, C*H*_2_, succinato), 2.59–2.64 (m, C*H*_2_, succinato), 2.27–2.34 (m, C*H*_2_, DACH), 2.07 (s, C*H*_3_), 2.05–2.11 (m, residues of acetylated dGC monomers), 1.52–1.71 (m, C*H*_2_, DACH), 1.22–1.32 (m, C*H*_2_, DACH) ppm. ^195^Pt NMR (D_2_O): δ = 3214 ppm.

10. Complex 6 Coupled to dGC P2 (C10)

The reaction was performed according to general procedure 3b using **6** (70 mg, 0.12 mmol, 1 eq), CDI (99 mg, 0.61 mmol, 5 eq), **P2** (50 mg, 0.24 mmol, 2 eq/monomer unit) and TEA (68 µL, 0.49 mmol, 4 eq). Yield: 88 mg. ICP-MS (Pt): 150.6 g/kg. ^1^H NMR (D_2_O): δ = 4.87 (b, O-C*H*-O, backbone of dGC), 3.59–4.11 (m, backbone of dGC), 3.18 (b, C*H*-NH/NH_2_, backbone of dGC), 2.84–2.95 (m, C*H*, DACH), 2.64–2.69 (m, C*H*_2_, succinato), 2.58–2.63 (m, C*H*_2_, succinato), 2.26–2.34 (m, C*H*_2_, DACH), 2.08 (s, C*H*_3_), 2.05–2.11 (m, residues of acetylated dGC monomers), 1.52–1.70 (m, C*H*_2_, DACH), 1.23–1.32 (m, C*H*_2_, DACH) ppm. ^195^Pt NMR (D_2_O): δ = 3214 ppm.

11. Complex 6 Coupled to dGC P2 (C11)

The reaction was performed according to general procedure 3a using **6** (70 mg, 0.12 mmol, 1 eq), CDI (103 mg, 0.63 mmol, 5 eq), **P2** (50 mg, 0.24 mmol, 2 eq/monomer unit) and TEA (64 µL, 0.46 mmol, 4 eq). Yield: 43 mg. ICP-MS (Pt): 200.3 g/kg. ^1^H NMR (D_2_O): δ = 4.87 (b, O-C*H*-O, backbone of dGC), 3.55–4.12 (m, backbone of dGC), 3.19 (b, C*H*-NH/NH_2_, backbone of dGC), 2.82–2.99 (m, C*H*, DACH), 2.64–2.75 (m, C*H*_2_, succinato), 2.58–2.64 (m, C*H*_2_, succinato), 2.25–2.36 (m, C*H*_2_, DACH), 2.07 (s, C*H*_3_), 2.05–2.11 (m, residues of acetylated dGC monomers), 1.53–1.71 (m, C*H*_2_, DACH), 1.19–1.32 (m, C*H*_2_, DACH) ppm. ^195^Pt NMR (D_2_O): δ = 3214 ppm.

12. Complex 6 Coupled to dGC P3 (C12)

The reaction was performed according to general procedure 3b using **6** (50 mg, 0.09 mmol, 1 eq), CDI (71 mg, 0.44 mmol, 5 eq), **P3** (72 mg, 0.35 mmol, 4 eq/monomer unit) and TEA (48 µL, 0.35 mmol, 4 eq). Yield: 99 mg. ICP-MS (Pt): 102.7 g/kg. ^1^H NMR (D_2_O): δ = 4.87 (b, O-C*H*-O, backbone of dGC), 3.54–4.12 (m, backbone of dGC), 3.18 (b, C*H*-NH/NH_2_, backbone of dGC), 2.84–2.95 (m, C*H*, DACH), 2.64–2.69 (m, C*H*_2_, succinato), 2.59–2.64 (m, C*H*_2_, succinato), 2.26–2.35 (m, C*H*_2_, DACH), 2.08 (s, C*H*_3_), 2.05–2.11 (m, residues of acetylated dGC monomers), 1.53–1.71 (m, C*H*_2_, DACH), 1.21–1.33 (m, C*H*_2_, DACH) ppm. ^195^Pt NMR (D_2_O): δ = 3213 ppm.

13. Complex 6 Coupled to dGC P1 (V1)

The reaction was performed according to general procedure 3a using **6** (71 mg, 0.12 mmol, 1 eq), CDI (41 mg, 0.25 mmol, 2 eq), dGC **P1** (50 mg, 0.24 mmol, 2 eq/monomer unit) and TEA (64 µL, 0.46 mmol, 4 eq). Yield: 43 mg. ICP-MS (Pt): 46.5 g/kg. ^1^H NMR (D_2_O): δ = 4.85 (b, O-C*H*-O, backbone of dGC), 3.55–4.12 (m, backbone of dGC), 3.18 (b, C*H*-NH/NH_2_, backbone of dGC), 2.85–2.96 (m, C*H*, DACH), 2.37–2.75 (m, C*H*_2_, succinato), 2.27–2.36 (m, C*H*_2_, DACH), 2.08 (b, C*H*_3_), 2.03–2.15 (m, residues of acetylated dGC monomers), 1.55–1.69 (m, C*H*_2_, DACH), 1.12–1.40 (m, C*H*_2_, DACH) ppm.

14. Complex 6 Coupled to dGC P2 (V2)

The reaction was performed according to general procedure 3a using **6** (72 mg, 0.13 mmol, 1 eq), CDI (41 mg, 0.25 mmol, 2 eq), dGC **P2** (50 mg, 0.24 mmol, 2 eq/monomer unit) and TEA (64 µL, 0.46 mmol, 4 eq). Yield: 39 mg. ICP-MS (Pt): 65.1 g/kg. ^1^H NMR (D_2_O): δ = 4.84 (b, O-C*H*-O, backbone of dGC), 3.54–4.13 (m, backbone of dGC), 3.17 (b, C*H*-NH/NH_2_, backbone of dGC), 2.86–2.97 (m, C*H*, DACH), 2.44–2.74 (m, C*H*_2_, succinato), 2.26–2.34 (m, C*H*_2_, DACH), 2.08 (b, C*H*_3_), 2.02–2.15 (m, residues of acetylated dGC monomers), 1.53–1.71 (m, C*H*_2_, DACH), 1.13–1.39 (m, C*H*_2_, DACH) ppm.

15. Complex 6 Coupled to dGC P3 (V3)

The reaction was performed according to general procedure 3a using **6** (70 mg, 0.12 mmol, 1 eq), CDI (41 mg, 0.25 mmol, 2 eq), dGC **P3** (50 mg, 0.24 mmol, 2 eq/monomer unit) and TEA (64 µL, 0.46 mmol, 4 eq). Yield: 38 mg. ICP-MS (Pt): 67.4 g/kg. ^1^H NMR (D_2_O): δ = 4.86 (b, O-C*H*-O, backbone of dGC), 3.52–4.12 (m, backbone of dGC), 3.18 (b, C*H*-NH/NH_2_, backbone of dGC), 2.86–2.97 (m, C*H*, DACH), 2.50–2.70 (m, C*H*_2_, succinato), 2.22–2.26 (m, C*H*_2_, DACH), 2.09 (b, C*H*_3_), 2.03–2.15 (m, residues of acetylated dGC monomers), 1.57–1.66 (m, C*H*_2_, DACH), 1.13–1.39 (m, C*H*_2_, DACH) ppm.

16. Complex 2 Coupled to dGC P3 (S1)

The reaction was performed according to general procedure 3a using **2** (55 mg, 0.11 mmol, 1 eq), CDI (98 mg, 0.60 mmol, 5 eq), dGC **P3** (47 mg, 0.23 mmol, 2 eq/monomer unit) and trimethylamine (64 µL, 0.46 mmol, 4 eq). Yield: 51 mg. ICP-MS (Pt): 88.7 g/kg. ^1^H NMR (D_2_O): δ = 3.50–4.10 (m, backbone of dGC), 3.14 (b, C*H*-NH/NH_2_, backbone of dGC), 2.56–2.75 (m, C*H*_2_, succinato), 2.08 (s, C*H*_3_), 1.99–2.07 (m, residues of acetylated dGC monomers) ppm.

17. Complex 2 Coupled to dGC P3 (S2)

The reaction was performed according to general procedure 3b using **2** (70 mg, 0.15 mmol, 1 eq), CDI (119 mg, 0.74 mmol, 5 eq), dGC **P3** (60 mg, 0.29 mmol, 2 eq/monomer unit) and trimethylamine (82 µL, 0.59 mmol, 4 eq). Yield: 35 mg. ICP-MS (Pt): 117.8 g/kg. ^1^H NMR (D_2_O): δ = 3.53–4.11 (m, backbone of dGC), 3.18 (b, C*H*-NH/NH_2_, backbone of dGC), 2.70–2.76 (m, C*H*_2_, succinato), 2.58–2.62 (m, C*H*_2_, succinato), 2.12 (s, C*H*_3_), 2.04–2.11 (m, residues of acetylated dGC monomers) ppm. ^195^Pt NMR (D_2_O): δ = 2705 ppm.

18. Complex 4 Coupled to dGC P3 (S3)

The reaction was performed according to general procedure 3b using **4** (150 mg, 0.30 mmol, 1 eq), CDI (240 mg, 1.48 mmol, 5 eq), dGC **P3** (122 mg, 0.59 mmol, 2 eq/monomer unit) and trimethylamine (165 µL, 1.19 mmol, 4 eq). Yield: 80 mg. ICP-MS (Pt): 91.9 g/kg. ^1^H NMR (D_2_O): δ = 3.54–4.01 (m, backbone of dGC), 3.10 (b, C*H*-NH/NH_2_, backbone of dGC), 2.45–2.72 (m, C*H*_2_, succinato + C*H*_2_-C, cyclobutyl), 2.03 (m, C*H*_2_, cyclobutyl) 2.00 (s, C*H*_3_), 1.93–2.09 (m, residues of acetylated dGC monomers) ppm.

19. Complex 4 Coupled to dGC P3 (S4)

The reaction was performed according to general procedure 3b using **4** (50 mg, 0.09 mmol, 1 eq), CDI (74 mg, 0.46 mmol, 5 eq), dGC **P3** (75 mg, 0.037 mmol, 4 eq/monomer unit) and trimethylamine (51 µL, 0.37 mmol, 4 eq). Yield: 92 mg. ICP-MS (Pt): 109.5 g/kg. ^1^H NMR (D_2_O): δ = 3.55–4.10 (m, backbone of dGC), 3.19 (b, C*H*-NH/NH_2_, backbone of dGC), 2.63–2.72 (m, C*H*_2_, succinato + C*H*_2_-C, cyclobutyl), 2.57–2.62 (m, C*H*_2_, succinato), 2.07 (s, C*H*_3_), 2.03 (m, C*H*_2_, cyclobutyl), 2.00–2.11 (m, residues of acetylated dGC monomers) ppm. ^195^Pt NMR (D_2_O): δ = 3508 ppm.

20. Complex 6 Coupled to dGC P3 (S5)

The reaction was performed according to general procedure 3b using **6** (71 mg, 0.12 mmol, 1 eq), CDI (99 mg, 0.61 mmol, 5 eq), **P3** (50 mg, 0.24 mmol, 2 eq/monomer unit) and TEA (68 µL, 0.49 mmol, 4 eq). Yield: 71 mg. ICP-MS (Pt): 88.7 g/kg. ^1^H NMR (D_2_O): δ = 4.87 (b, O-C*H*-O, backbone of dGC), 3.52–4.15 (m, backbone of dGC), 3.19 (b, C*H*-NH/NH_2_, backbone of dGC), 2.84–2.96 (m, C*H*, DACH), 2.64–2.69 (m, C*H*_2_, succinato), 2.59–2.64 (m, C*H*_2_, succinato), 2.27–2.34 (m, C*H*_2_, DACH), 2.08 (s, C*H*_3_), 2.04–2.12 (m, residues of acetylated dGC monomers), 1.54–1.70 (m, C*H*_2_, DACH), 1.21–1.33 (m, C*H*_2_, DACH) ppm. ^195^Pt NMR (D_2_O): δ = 3214 ppm. 

### 2.6. ICP-MS

Platinum(IV)–dGC conjugates (0.5–1.5 mg) were digested in 2 mL of HNO_3_ (20%) and 0.1 mL of H_2_O_2_ (30%) with a temperature-controlled heating plate of graphite from Labter. Afterwards the samples were diluted (1:10,000) with HNO_3_ (3%), and the total platinum concentration was detected with an Agilent 7800 ICP-MS instrument using rhenium as an internal reference. An Agilent SPS 4 autosampler was used to carry out 10 replicates for each sample, and the data were evaluated with the Agilent MassHunter software. 

### 2.7. Cell Culture

Cell culture of the cell lines CH1/PA-1, SW480 and A549 was performed as described in reference [38]. 4T1 cells were purchased from American Type Culture Collection, stored in a liquid nitrogen dewar and cultured in Roswell Park Memorial Institute (RPMI)-1640 medium supplemented with 10% foetal bovine serum (FBS) (Thermo Fisher, Waltham, MA, USA).

### 2.8. Cytotoxicity

Cytotoxicity tests (96 h incubation) in the cell lines CH1/PA-1, SW480 and A549 were conducted as described in reference [38]. Compounds were dissolved in supplemented minimum essential medium (MEM), except for compound **C10,** which was dissolved in sterile Milli-Q water.

To seed 4T1 cells at a density of 1 × 10^4^ cells per well, 96-well plates were used. 4T1 cells were incubated at 37 °C in 5% CO_2_, 95% in air for 24 h in Roswell Park Memorial Institute (RPMI)-1640 medium supplemented with 10% foetal bovine serum (FBS). Just before treatment, the medium was removed, and the cells were washed twice with phosphate buffered saline (PBS, pH = 7.4). Conjugates **V1**, **V2** and **V3** (1 mg/mL) were suspended in Hank’s Balanced Salt Solution (HBSS) and 200 μL volume was dispensed into each well and left for 2 h. HBSS was used as the negative control, while a 1% Triton X-100 solution in PBS was used as the positive control. The treatment was subsequently removed by pipetting and the cells were washed twice with PBS. A stock solution of the MTT reagent (5 mg/mL) was prepared by suspending in PBS, after which a 0.5 mg/mL working solution was prepared from the stock; then, 200 μL of the working solution was transferred into each well, and the plate was incubated until the formed purple formazan crystals could be detected by means of a microscope (typically 2 h). The MTT reagent was subsequently removed from the wells by pipetting, and the crystals were dissolved by adding 200 μL of DMSO to each well and shaking on an orbital shaker for 15 min, protected from light. The plates were subsequently read on an UV spectrophotometer at a wavelength of 570 nm. Cell viability was further determined as a percentage of the negative control (HBSS).

### 2.9. Biodistribution Study: 1 h and 24 h

The Animal Welfare and Ethical Review Body at University College London (UCL) authorised all animal experiments, which were performed under license from the Home Office, UK. All animals were kept in a pathogen-free environment and treated in accordance with the Institutional Committees on Animal Welfare of the U.K. Home Office (the Home Office Animals Scientific Procedures Act, 1986). Biodistribution studies on a platinum-loaded polymer **V3** compared to the platinum(IV) complex **6** were carried out, using female Balb/C mice (20–22.5 g) (Harlan, UK) divided into 2 time points (t_1_ = 1 h, t_2_ = 24 h, 4 groups n = 5). The used concentrations of 0.41 mg/100 µL for compound **V3** and 0.15 mg/100 µL for complex **6** are equivalent to 2.75 mg/kg oxaliplatin and 5.5 mg/kg, respectively. All substances were applied intravenously (100 µL stock/20 g body weight) via tail vein injection. At the chosen time points, animals were culled with CO_2,_ and the major organs (lung, heart, liver, spleen and kidneys) were collected and frozen in liquid nitrogen. The samples were transferred to the Institute of Inorganic Chemistry, University of Vienna, Austria, where the total platinum content of each sample was determined with an Agilent 7500 ICP-quadrupole MS instrument. 

## 3. Results and Discussion

### 3.1. Synthesis

**Scheme 3:**
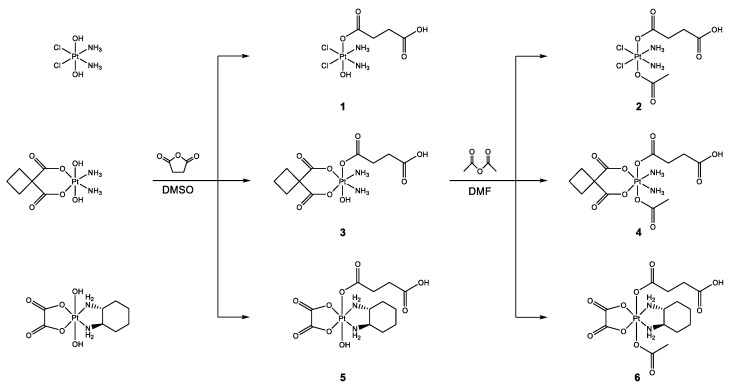
Synthetic pathway for platinum(IV) complexes **1**–**6** with mixed axial ligands and a free carboxylic acid group to enable the introduction of drug delivery platforms.

Previously published synthetic routes served as a template and were modified for the synthesis of platinum(IV) complexes **1**–**6** with a mixed axial ligand sphere (Scheme 3) [13,36]. 

Starting from the corresponding dihydroxidoplatinum(IV) complex, compounds **1**, **3**, and **5** were synthesised via carboxylation with succinic anhydride in absolute DMSO. Afterwards, the addition of acetic anhydride in absolute DMF and purification by preparative RP-HPLC resulted in the final products **2**, **4**, and **6**. 

**Scheme 4:**
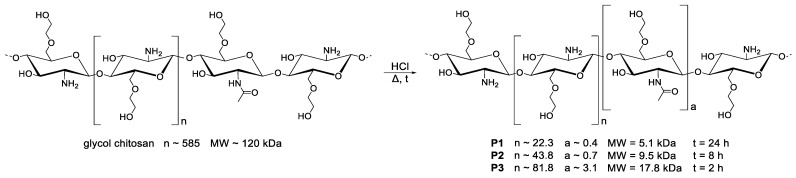
Time-dependent acidic degradation of glycol chitosan results in dGC polymers with different molecular weights (MW) **P1**–**P3**. The remaining degree of acetylation was determined with ^1^H NMR spectroscopy and was detected as about 1% for **P1**, 2% for **P2**, and 4% for **P3**.

Glycol chitosan polymer chains (MW~120 kDa) were degraded by using hydrochloric acid followed by time-dependent incubation at 50 °C. As longer degradation times lead to shorter chain lengths, the following three time points were chosen: 2, 8, and 24 h to obtain MW~18 kDa (**P3**), MW~10 kDa (**P2**), and MW~5 kDa (**P1**), respectively. Afterwards, the solutions were freeze-dried to yield white fibrous solids (Scheme 4).

**Scheme 5:**
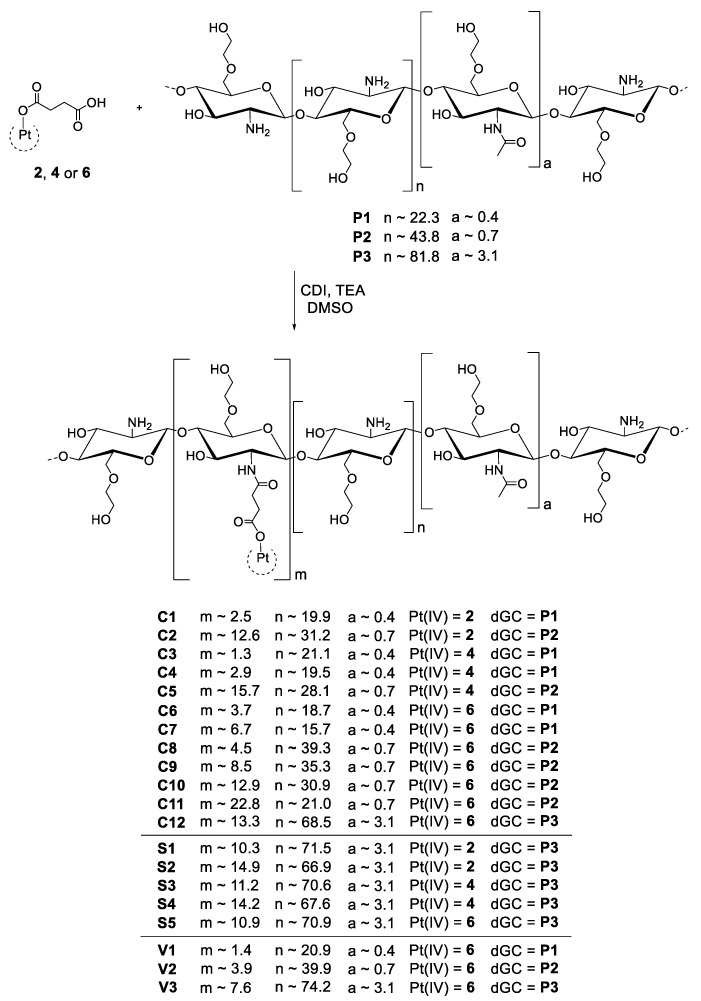
Scheme of CDI coupling reaction of the unsymmetric platinum(IV) complexes and dGC polymers, yielding 20 conjugates with a platinum(IV) to dGC polymer ratio between 1.3 and 22.8.

In particular, unsymmetrically carboxylated platinum(IV) complexes are in accordance with an optimised reduction potential preventing premature reduction to the corresponding platinum(II) species [39]. The free carboxylic acid group of complexes **2**, **4_,_** and **6** in axial position enables the attachment to the primary amine of the dGC polymer via amide bond formation. The coupling reaction was thereby performed in the presence of the peptide coupling reagent 1,1’-carbonyldiimidazole (CDI). First, the carboxylic acid moiety of compound **2**, **4**, or **6** was activated with CDI, resulting in the reactive imidazolide. In parallel, CO_2_ and imidazole were released. Afterwards, the activated carboxylic acid was brought to reaction with the NH_2_ moiety of the dGC polymers, leading to amide bond formation [40,41]. Afterwards, the conjugates (**C1**–**C12**, **S1**–**S5**, **V1**–**V3**) were purified via dialysis against distilled water and 0.1 M hydrochloric acid, in succession, in order to remove unreacted CDI, uncoupled platinum(IV) complexes and imidazole. Finally, the conjugates were freeze-dried and obtained as whitish fibrous solids (Scheme 5).

In addition to CDI, varying coupling reagents as well as different solvents and complex to polymer ratios were evaluated. Overall, the best results were observed in DMSO with maximum 0.5 equivalents of the corresponding CDI-activated platinum(IV) complex. The complex:polymer ratio of a maximum of 1:2 is crucial for the solubility, especially with the longest dGC polymer **P3**. In addition to conjugate **C12**, another conjugation of complex **6** and dGC polymer **P3** (**S5**) as well as the coupling of complex **2** and **4** to **P3** were successfully performed (**S1**–**S4**) with complex:polymer ratios of 1:2 and 1:4, respectively. Based on the best solubility, a series of conjugates (**V1**–**V3**) with oxaliplatin(IV) analogue **6** and all three dGC batches (**P1**–**P3**) were synthesised de novo for subsequent in vivo experiments. In addition to lower complex to polymer ratios, acidic conditions before freeze-drying affected the solubility. As a result of the change from distilled water to 0.1 M hydrochloric acid at the end of the dialysis process, the adjustment to pH 3 significantly improved the solubility of the product, possibly due to the formation of amine salts. Finally, a continuous decline of solubility was detected by conjugates over time. However, the solubility loss could be substantially decelerated by storage of the conjugates under an argon atmosphere at −30 °C. 

### 3.2. Analysis

The final platinum(IV) complexes **2**, **4**, and **6** were characterised with one- and two-dimensional NMR spectroscopy (^1^H, ^13^C, ^15^N, ^195^Pt) (Supporting Information, Figures S1–S9), and their purity (>95%) was confirmed by elemental analysis. 

The molecular weight of the different dGC batches **P1**–**P3** was determined by gel permeation chromatography with multi-angle laser light scattering (GPC-MALLS); the results are presented in Table 1. Additionally, one representative NMR spectrum of the dGC polymer can be found in the Supporting Information (Figure S10).

The characterisation of the conjugates **C1**–**C12** was performed with ^1^H and ^195^Pt NMR spectroscopy, proving the presence of the corresponding platinum(IV) complexes (Supporting Information, Figures S13–S18). The ^195^Pt NMR spectra showed characteristic signals for platinum(IV) species between 2700 and 3500 ppm. Especially, the superimposition of the ^1^H NMR spectra of the platinum(IV) analogue of oxaliplatin **6** and the unloaded dGC polymer highlights the additional peaks in the upfield region between 1.0 and 3.0 ppm of the NMR spectra of the corresponding conjugates (Supporting Information, Figure S17). 

The average levels of platinum(IV) complexes per dGC polymer for all conjugates were determined by means of inductively coupled plasma MS (ICP-MS) and were in the range of 5.4% to 49.8%, equivalent to 1.3–22.8 platinum(IV) units per polymer molecule. The obtained platinum(IV) units per dGC polymer molecule were used for the calculation of the molecular weight of all conjugates (Table 2), and the amount of platinum(IV) units affected the solubility. In general, lower platinum(IV) loading levels, and the resulting smaller molecular weights resulted in better solubility. This relationship is particularly distinct for conjugates with the same dGC polymer, platinum(IV) complex and molecular weights ~15 kDa. Conjugates with molecular weights ~20 kDa, mainly achieved with **P3**, showed the lowest solubility of all conjugates. Additionally, solubility differences between conjugates containing different dGC polymers and platinum(IV) complexes were identified in the following order, starting with the best solubility: **P1** > **P2** > **P3** and **6** > **4** > **2** (Supporting Information, Table S1). 

### 3.3. Cytotoxicity

Based on the required solubility, conjugates **C1**–**C12** were forwarded for cytotoxicity assessments, whereas conjugates **S1**–**S5** could not be tested due to solubility issues (Supporting Information, Table S1). The twelve substances **C1**–**C12** were examined in comparison to the free platinum(IV) complexes and the unloaded dGC polymers with three different chain lengths for their cytotoxic activity in vitro. For this purpose, the colorimetric MTT assay was used in three human cancer cell lines (A549 (non-small-cell lung carcinoma), CH1/PA-1 (ovarian teratocarcinoma) and SW480 (colon adenocarcinoma)), yielding IC_50_ values in the low micromolar to nanomolar concentration range for all conjugates (Table 3, Supporting Information, Figures S18–S20). The three tested cell lines differ in their platinum sensitivity. SW480 and A549 cells are intrinsically cisplatin- and carboplatin-resistant due to their low expression of CTR1, whereas CH1/PA-1 cells show high chemosensitivity, particularly to platinum drugs [42,43]. 

In general, platinum(IV) complexes exert even slower biological effects than their platinum(II) analogues; consequently, complexes **2**, **4**, and **6** exhibited lower cytotoxic potencies than their platinum(II) counterparts. The dGC polymers **P1**, **P2**, and **P3** showed negligible cytotoxicity within 96 h, with IC_50_ values higher than 100 µM, with the exception of **P3,** displaying an IC_50_ value of 77 ± 25 µM in CH1/PA-1 cells.

**Figure 1:**
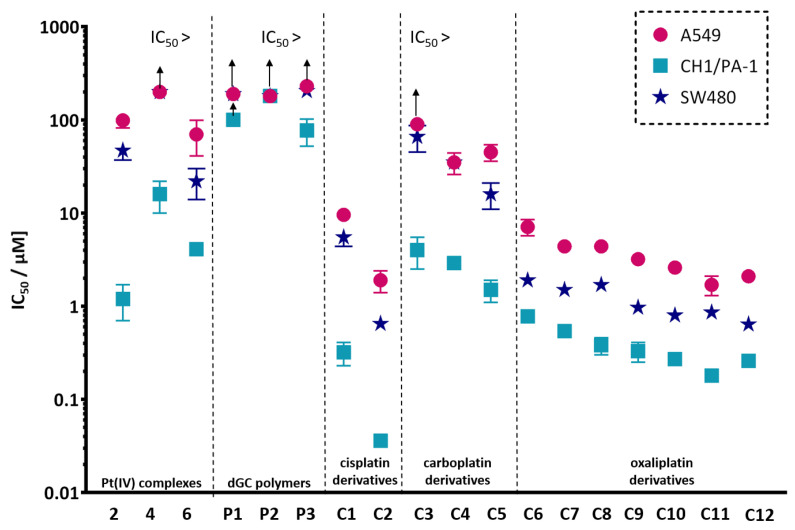
Structure–activity relationship of the studied platinum(IV) complexes, dGC polymers and conjugates. The y-axis represents IC_50_ values (means ± standard deviations) in all three cell lines on a logarithmic scale. The arrows indicate IC_50_ values higher than the indicated value.

On the contrary, conjugates (**C1**–**C12**) revealed different antiproliferative activities in the three tested cell lines, exhibiting the highest activity against the commonly most sensitive cell line CH1/PA-1, moderate activity in the cell line SW480, and the lowest activity in the intrinsically more chemoresistant A549 cell line (Figure 1). 

Remarkably, the most potent conjugate **C2**, with IC_50_ values of 0.036 ± 0.005 µM (CH1/PA-1), 1.9 ± 0.5 µM (A549) and 0.65 ± 0.07 µM (SW480), was two times more active in CH1/PA-1 and A549 cells and three-and-a-half times more active in the cell line SW480 than its platinum(II) counterpart cisplatin. Compared with the corresponding platinum(IV) complex **2**, **C2** demonstrated an enormous increase in cytotoxicity by factors of 33 (CH1/PA-1), 52 (A549) and 72 (SW480), based on IC_50_ values. The second most cytotoxic compound was **C11**, containing the highest loading with platinum(IV) units (22.8 per polymer) of all examined conjugates. In contrast to **C2**, **C11** did not reveal a higher antiproliferative effect than the platinum(II) counterpart (oxaliplatin). However, it displayed significantly enhanced cytotoxicity compared to the corresponding platinum(IV) complex **6** by factors of 22 (CH1/PA-1), 25 (SW480), and 41 (A549). Conjugate **C3** yielded the lowest antiproliferative activity of all conjugates, in line with the overall highest IC_50_ value of carboplatin(IV) analogue **4** and lowest loading with platinum(IV) units (1.3 per polymer molecule). 

Within the set of all dGC polymers bearing platinum(IV) complexes, cisplatin(IV)-containing conjugates **C1** and **C2** were more favourable in terms of antiproliferative activity, correlating very well with the high cytotoxicity of cisplatin. **C1** appeared less potent, containing five times less platinum(IV) units than **C2**, which revealed the highest cytotoxic potency of all conjugates. Both, **C1** and **C2** showed higher antiproliferative activity in all tested cell lines than their platinum(IV) analogue **2**.

The conjugate group of carboplatin(IV) derivatives **C3**–**C5** featured the highest IC_50_ values, consistent with the low cytotoxicity of carboplatin(IV) complex **4**. **C3** and **C4**, both coupled to **P1**, differ in their amount of conjugated platinum(IV) units, demonstrating a clear correlation with cytotoxicity. Expectedly, the higher platinum(IV) loading of **C4** resulted in lower IC_50_ values compared to **C3**. Otherwise, **C5** exhibited the lowest IC_50_ values in CH1/PA-1 and SW480 cells within this group of conjugates, likely correlated with a higher platinum(IV) loading level. Collectively, all three conjugates **C3**–**C5** revealed enhanced antiproliferative activity compared to the corresponding platinum(IV) complex **4**. 

Compounds **C6**–**C12**, featuring oxaliplatin analogues in the oxidation state IV, covered a wide cytotoxic activity span, with IC_50_ values ranging from 0.18 µM to 7.1 µM. The above-mentioned correlation, based on the number of coordinated platinum(IV) units leading to higher cytotoxicity, was further observed within groups of the same chain lengths. Accordingly, **C7** displayed advantageously lower IC_50_ values in all cell lines than **C6**. The dGC polymer **P2,** containing series **C8**–**C11,** showed different amounts of platinum(IV) units, ranging from 4.5 to 22.8 per polymer molecule, showing again a pronounced dependency of cytotoxicity on the number of platinum(IV) units. Therefore, **C8** exhibited the lowest, whereas **C11** displayed the highest antiproliferative activity in all cell lines. The only exception to this pattern was observed for **C11**, with a higher IC_50_ value in SW480 cells. Due to low solubility of conjugate **S5**, **C12** was the only tested compound based on dGC polymer **P3**, and it revealed one of the highest antiproliferative effects. **C10** and **C12**, with a negligible difference in amount of platinum(IV) units and thus mainly differing in the polymer carrier, revealed comparable IC_50_ values in each and every cell line. On the contrary, the correlation of platinum(IV) loading and higher cytotoxicity was not as pronounced for conjugates with different dGC batches, such as **C7** and **C8**. Consequently, the polymer chain length has a minor effect on the cytotoxic activity. 

**Figure 2:**
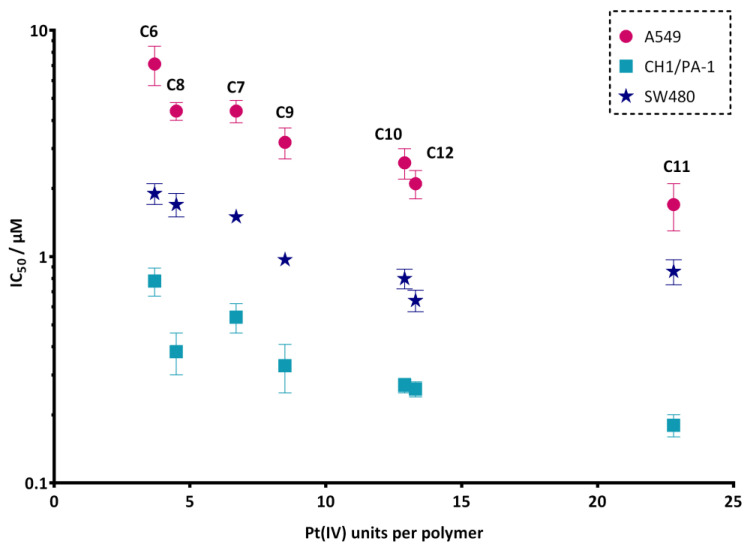
Correlation between IC_50_ values and platinum(IV) loading of oxaliplatin derivatives (**C6**–**C12**). The y-axis represents IC_50_ values (means ± standard deviations) in all three cell lines on a logarithmic scale; the x-axis is related to the platinum(IV) units per polymer.

Additionally, the correlation between IC_50_ values and platinum(IV) load of oxaliplatin derivatives (**C6**–**C12**) was studied and is shown in Figure 2. In all three cell lines, the same trend could be observed: the higher the platinum(IV) load, the lower the IC_50_ value of the tested conjugates. An unpaired *t*-test with Welch’s correction was performed between the most (**C11**) and the least (**C6**) cytotoxic complexes, revealing significant differences, with *p* < 0.05 (A549), *p* < 0.01 (CH1/PA-1) and *p* < 0.01 (SW480).

In order to further investigate the influence of conjugation on the cytotoxicity, the concentration levels of the corresponding platinum(IV) units at the IC_50_ value of conjugates **C1**–**C12** and **V1**–**V3** were calculated (Table 4). The comparison of the normalised IC_50_ values with the IC_50_ values of the uncoupled platinum(IV) complexes **2**, **4**, **6** displayed an evident increase in cytotoxicity through conjugation. This connection could be observed for all conjugates except for **C5** in all cell lines. Depending on the coupled platinum(IV) complex and the cell line, the factors for increased cytotoxicity varied between 1.0 and 5.7, following the trend **4** < **6** < **2** and CH1/PA-1 < A549 ≈ SW480. 

Based on these observations, it can be concluded that in general, conjugation of platinum(IV) complexes to dGC polymers led to increased cytotoxicity. Furthermore, higher loading with platinum(IV) units consistently enhanced the antiproliferative activity within the group of the same dGC polymer. On the other hand, no systematic structure–activity relationship could be observed with regard to conjugates differing in the chain length of dGC. Overall, conjugates with lower chain lengths and high platinum(IV) loading levels are the most promising candidates, based on good solubility combined with high potency. Moreover, all conjugates showed enhanced cytotoxicity compared to the corresponding platinum(IV) complexes. 

Based on the highly promising results, we chose to push the most active compounds offering favourable solubility forward, based on dGC polymer chain length and platinum core. Despite accurate controlling of the reaction parameters, it remains a challenge to precisely control the platinum(IV) load. For animal experiments, oxaliplatin(IV)-based conjugates **V1**–**V3** were resynthesised with 1.4, 3.9, and 7.6 units of platinum(IV) per dGC polymer molecule, respectively. Their cytotoxicity was investigated in murine mammary carcinoma cell line 4T1, used for the in vivo experiments in mice. In general, IC_50_ values in the low micromolar range were revealed, and these results emphasise the previously examined structure–activity relationships of platinum(IV) loading levels and cytotoxic activity, with **V1** exhibiting the lowest antiproliferative effect, whereas **V3** was the most active conjugate (Table 5). 

### 3.4. Biodistribution Studies

**Figure 3:**
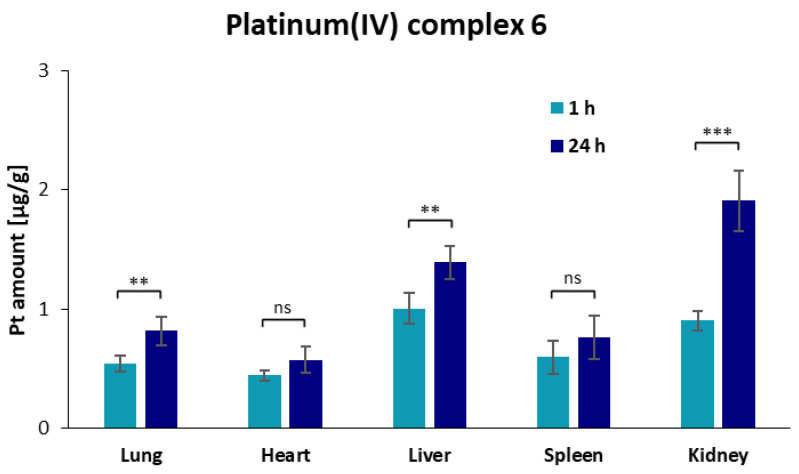

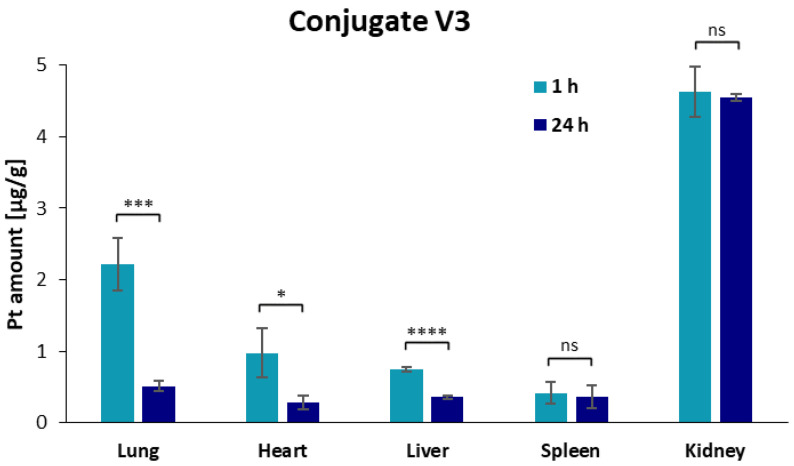
Overview of the accumulation of platinum in different organs, measured with ICP-MS, as result of the biodistribution studies for platinum(IV) complex **6** and conjugate **V3** after 1 h and 24 h after intravenous administration. Additionally, significance of the increase and decrease in the platinum amount between 1 h and 24 h of complex **6** (upper figure) and conjugate **V3** (lower figure) was further examined via unpaired *t*-test with Welch*’*s correction, with the following abbreviations: ns = not significant; * *p* < 0.05; ** *p* < 0.01; *** *p* < 0.001; **** *p* < 0.0001.

Conjugate **V3**, offering both high cytotoxicity and sufficient solubility, was investigated for its biodistribution in vivo in comparison to its respective free platinum(IV) complex **6**. Intravenously, 0.15 mg/100 µL of platinum(IV) complex **6** and 0.41 mg/100 µL of conjugate **V3** were administered to five non-tumour-bearing female Balb/C mice per group. The used concentrations are equivalent to 5.5 mg/kg and 2.75 mg/kg oxaliplatin, respectively, and were well tolerated without signs of toxicity or any other negative impact on mouse health. The major organs of liver, lung, heart, kidney and spleen were collected after two specific time points (1 h and 24 h), and the platinum content of each organ was evaluated with ICP-MS analysis (Figure 3).

The free platinum(IV) complex **6** was similarly distributed in lung, heart, and spleen and displayed the highest concentrations in the excretory organs (kidney and liver) 1 h post administration. After 24 h, the platinum amount was only slightly raised in all systemic organs. The highest increase of 2.1-fold was in the kidneys, as indication of excretion. The high platinum levels in the kidney are in accordance with previously published data of an oxaliplatin(IV) analogue and other platinum(IV) complexes [44,45]. However, complex **6** displayed significantly lower platinum amounts in the liver, possibly caused by the lower lipophilicity of compound **6**.

On the contrary, conjugate **V3** showed the highest accumulation in the kidneys, followed by the lungs and heart, and the lowest amounts in the liver and spleen after 1 h, indicating higher renal excretion of the conjugates compared to complex **6**. Platinum concentrations decreased in all organs for conjugate **V3** 24 h post injection but remained high in the kidney. These results are in accordance with previously published studies of dGC polymers loaded with other chemotherapeutics, such as doxorubicin [46,47]. The renal uptake is characteristic for glycol chitosan polymers based on their ability to bind to the megalin receptor in tubular cells of the kidney [48,49,50]. Cisplatin-associated nephrotoxicity is not reported for oxaliplatin [51], and together with the nontoxicity of glycol chitosan polymers [49], a safe pharmacokinetic profile for conjugate **V3** can be expected. Additionally, the biodistribution study of conjugate **V3** revealed an increased accumulation in lung tissue compared to complex **6** and other platinum(IV) complexes [44,45]. 

Finally, the altered kinetic profile of platinum(IV) complexes through coupling to dGC polymers reveals a promising approach for future cancer research, maybe also with respect to cancer of the lung or lung metastases. 

## 4. Conclusions

The synthesis of a series of platinum(IV) analogues of cisplatin, carboplatin and oxaliplatin coordinated to dGC polymers with different molecular weights resulted in 15 conjugates with various average platinum(IV) loading levels. In vitro cytotoxicity assays displayed promising cytotoxic activity in human cancer cell lines, with IC_50_ values in the low micromolar to nanomolar range. All conjugates revealed lower IC_50_ values than the corresponding unconjugated platinum(IV) complex and were comparable with the platinum(II) counterparts. Generally, it can be concluded that conjugation of platinum(IV) complexes to dGC polymers led to increased cytotoxicity. Additionally, a tendency with higher amounts of platinum(IV) units per dGC polymer leading to lower IC_50_ values could be observed. One oxaliplatin(IV)–dGC conjugate was further investigated in a biodistribution experiment in non-tumour-bearing Balb/C mice, revealing an enhanced accumulation in the lung compared to the free oxaliplatin(IV) analogue. Consequently, these results imply a great potential for a novel anticancer treatment, especially with regard to lung cancer types and lung metastases. Therefore, activity studies in tumour-bearing mice would be the next step in order to evaluate the promising potential of platinum(IV)–dGC conjugates. 

## Figures and Tables

**Table 1:** Overview of the different molecular weights of the dGC polymers **P1**–**P3**.

dGC Batch	Degradation Time (h)	MW (kDa)
**P1**	24	5.074
**P2**	8	9.539
**P3**	2	17.830

**Table 2:** Overview of the platinum(IV) units per dGC polymer molecule and average loading levels of conjugates **C1**–**C12**, **S1**–**S5**, **V1**–**V3**.

Sample	Pt (IV) Precursor	dGC	Monomer Units *	Pt Loading [%] **	MW [kDa] Unloaded	MW [kDa] Loaded
**C1**	**2**	**P1**	24.3	10.3	5.1	6.6
**C2**	**2**	**P2**	45.8	27.5	9.5	15.7
**C3**	**4**	**P1**	24.3	5.4	5.1	6.2
**C4**	**4**	**P1**	24.3	11.9	5.1	7.0
**C5**	**4**	**P2**	45.8	34.3	9.5	18.3
**C6**	**6**	**P1**	24.3	15.2	5.1	7.5
**C7**	**6**	**P1**	24.3	27.6	5.1	9.2
**C8**	**6**	**P2**	45.8	9.8	9.5	12.5
**C9**	**6**	**P2**	45.8	18.6	9.5	14.7
**C10**	**6**	**P2**	45.8	28.2	9.5	17.1
**C11**	**6**	**P2**	45.8	49.8	9.5	22.6
**C12**	**6**	**P3**	83.8	15.9	17.8	25.6
**S1**	**2**	**P3**	83.8	12.3	17.8	22.9
**S2**	**2**	**P3**	83.8	17.8	17.8	25.1
**S3**	**4**	**P3**	83.8	13.4	17.8	24.2
**S4**	**4**	**P3**	83.8	16.9	17.8	25.8
**S5**	**6**	**P3**	83.8	13.0	17.8	24.3
**V1**	**6**	**P1**	24.3	5.8	5.1	6.3
**V2**	**6**	**P2**	45.8	8.5	9.5	12.1
**V3**	**6**	**P3**	83.8	9.1	17.8	22.5

* Amount of monomer units without acetylated monomer units. ** Referred to monomer units without acetylation.

**Table 3:** IC_50_ values of conjugates **C1**–**C12** in comparison to platinum(II) and free platinum(IV) complexes (**2**, **4**, **6**) as well as to unloaded dGC polymers (**P1**–**P3**) in the human cancer cell lines A549, CH1/PA-1 and SW480.

Sample	Pt(IV)	dGC	Pt(IV) Units per Polymer	IC_50_ [µM] A549	IC_50_ [µM] CH1/PA-1	IC_50_ [µM] SW480
Cisplatin [38]	-		-	3.8 ± 1.0	0.073 ± 0.001	2.3 ± 0.2
Carboplatin [38]	-		-	38 ± 3	0.79 ± 0.11	42 ± 10
Oxaliplatin [38]	-		-	0.98 ± 0.21	0.18 ± 0.01	0.29 ± 0.05
**2**	**2**		-	99 ± 17	1.2 ± 0.5	47 ± 10
**4**	**4**		-	>200	16 ± 6	>200
**6**	**6**		-	70 ± 29	4.1 ± 0.6	22 ± 8
**P1**	-	**P1**	-	>190	>100	>190
**P2**	-	**P2**	-	>180	>180	>180
**P3**	-	**P3**	-	>230	77 ± 25	205 ± 16
**C1**	**2**	**P1**	2.5	9.6 ± 0.7	0.32 ± 0.09	5.5 ± 1.1
**C2**	**2**	**P2**	12.6	1.9 ± 0.5	0.036 ± 0.005	0.65 ± 0.07
**C3**	**4**	**P1**	1.3	>90	4.0 ± 1.5	66 ± 21
**C4**	**4**	**P1**	2.9	35 ± 9	2.9 ± 0.4	35 ± 5
**C5**	**4**	**P2**	15.7	45 ± 9	1.5 ± 0.4	16 ± 5
**C6**	**6**	**P1**	3.7	7.1 ± 1.4	0.78 ± 0.11	1.9 ± 0.2
**C7**	**6**	**P1**	6.7	4.4 ± 0.5	0.54 ± 0.08	1.5 ± 0.1
**C8**	**6**	**P2**	4.5	4.4 ± 0.4	0.38 ± 0.08	1.7 ± 0.2
**C9**	**6**	**P2**	8.5	3.2 ± 0.5	0.33 ± 0.08	0.97 ± 0.06
**C10**	**6**	**P2**	12.9	2.6 ± 0.4	0.27 ± 0.02	0.80 ± 0.08
**C11**	**6**	**P2**	22.8	1.7 ± 0.4	0.18 ± 0.02	0.86 ± 0.11
**C12**	**6**	**P3**	13.3	2.1 ± 0.3	0.26 ± 0.02	0.64 ± 0.07

**Table 4:** Overview of the concentration levels of platinum(IV) unit at the IC_50_ value calculated on the basis of the measured IC_50_ values of conjugates **C1**–**C12** and **V1**–**V3** in the corresponding cancer cell lines.

		Concentration of Pt(IV) Units at IC_50_ [µM]			
Sample	Pt(IV)	A549	CH1/PA-1	SW480	4T1
**2**	**2**	99 ± 17	1.2 ± 0.5	47 ± 10	-
**4**	**4**	>200	16 ± 6	>200	-
**6**	**6**	70 ± 29	4.1 ± 0.6	22 ± 8	-
**C1**	**2**	24	0.80	14	-
**C2**	**2**	24	0.45	8.2	-
**C3**	**4**	117	5.2	86	-
**C4**	**4**	102	8.4	102	-
**C5**	**4**	707	24	251	-
**C6**	**6**	26	2.9	7.0	-
**C7**	**6**	29	3.6	10	-
**C8**	**6**	20	1.7	7.7	-
**C9**	**6**	27	2.8	8.2	-
**C10**	**6**	34	3.5	10	-
**C11**	**6**	39	4.1	20	-
**C12**	**6**	28	3.5	8.5	-
**V1**	**6**	-	-	-	25
**V2**	**6**	-	-	-	41
**V3**	**6**	-	-	-	28

**Table 5:** IC_50_ values of conjugates **V1**–**V3** with the platinum(IV) analogue of oxaliplatin in the murine mammary carcinoma cell line 4T1.

Sample	Pt(IV)	dGC	Pt(IV) Units per Polymer	IC_50_ [µM] 4T1
**V1**	**6**	**P1**	1.4	18.0 ± 6.4
**V2**	**6**	**P2**	3.9	10.4 ± 3.0
**V3**	**6**	**P3**	7.6	3.7 ± 1.3

## Data Availability

The data presented in this study are available in the supplementary material.

## Supplementary Materials

The following supporting information can be downloaded at https://www.mdpi.com/article/10.3390/pharmaceutics15041050/s1, Figures S1–S9: NMR spectra of platinum(IV) complexes; Figure S10: NMR spectra of dGC polymers; Figures S11–S17: NMR spectra of conjugates; Figures S18–S20: Concentration–effect curves; Table S1: solubility data.
