# Supplementary material for: Platinum(IV)-Loaded Degraded Glycol Chitosan as Efficient Platinum(IV) Drug Delivery Platform
Source: Pharmaceutics. 2023 Mar 24;15(4):1050. doi: 10.3390/pharmaceutics15041050 (PMC10145531; doi:10.3390/pharmaceutics15041050)
Supplement: Supplementary file 1 [file pharmaceutics-15-01050-s001.zip › pharmaceutics-2227524-supplementary.pdf]

Supporting Information

Platinum(IV) loaded degraded glycol chitosan as efficient platinum(IV) drug delivery platform

Yvonne Lerchbammer-Kreith [1,†], Nadine S. Sommerfeld [1,†], Klaudia Cseh [1], Xian Weng-Jiang [2], Uchechukwu Odunze [2], Andreas G. Schätzlein [2], Ijeoma F. Uchegbu [2], Mathea S. Galanski [1], Michael A. Jakupec [1,3,*] and Bernhard K. Keppler [1,3]

1 Institute of Inorganic Chemistry, Faculty of Chemistry, University of Vienna, Waehringer Strasse 42, 1090 Vienna, Austria
2 School of Pharmacy, University College London, Brunswick Square 29-39, London WC1N 1AX, UK
3 Research Cluster "Translational Cancer Therapy Research", University of Vienna, Waehringer Strasse 42, 1090 Vienna, Austria
* Correspondence: michael.jakupec@univie.ac.at
† These authors contributed equally to this work.

Table of Contents

1. **NMR spectra of platinum(IV) complexes** 2
2. **NMR spectra of dGC polymers** 8
3. **NMR spectra of conjugates** 9
4. **Concentration-effect curves** 13
5. **Solubility data** 16

1. NMR spectra of platinum(IV) complexes

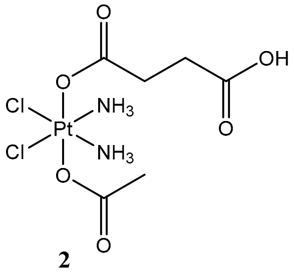

2

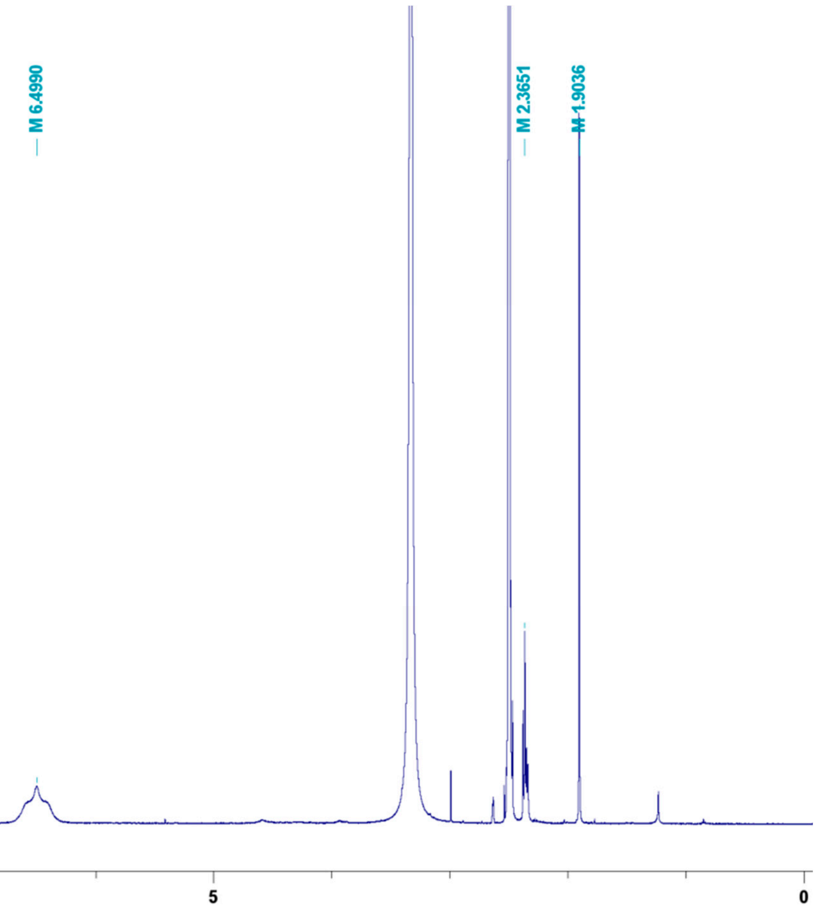

Figure S1. ^{1}H NMR spectrum of complex **2** in d_6-DMSO.

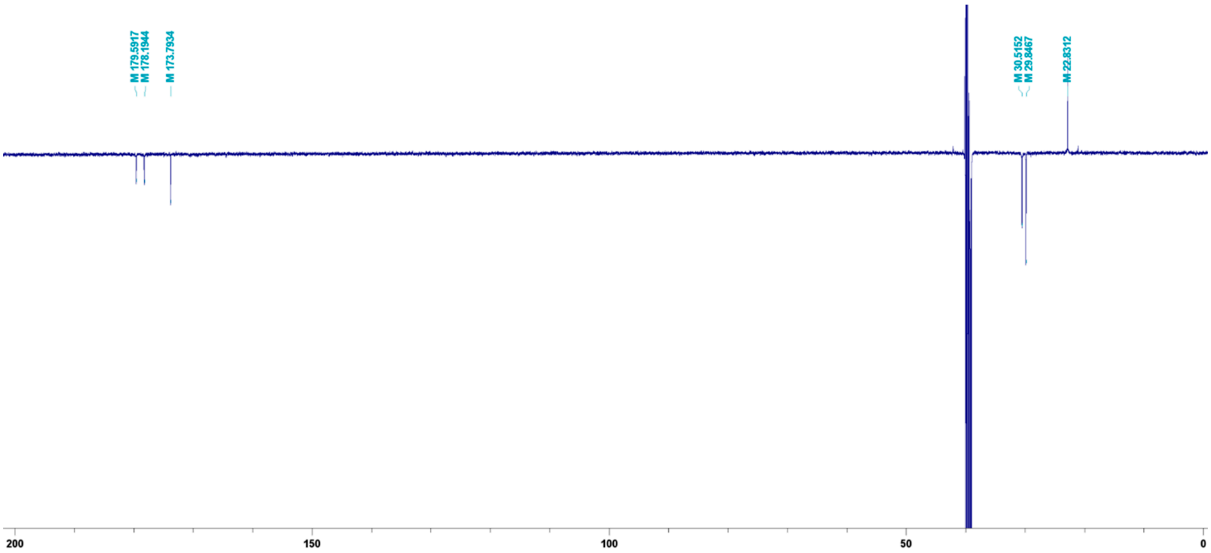

Figure S2. ^{13}C NMR spectrum of complex **2** in d_6-DMSO.

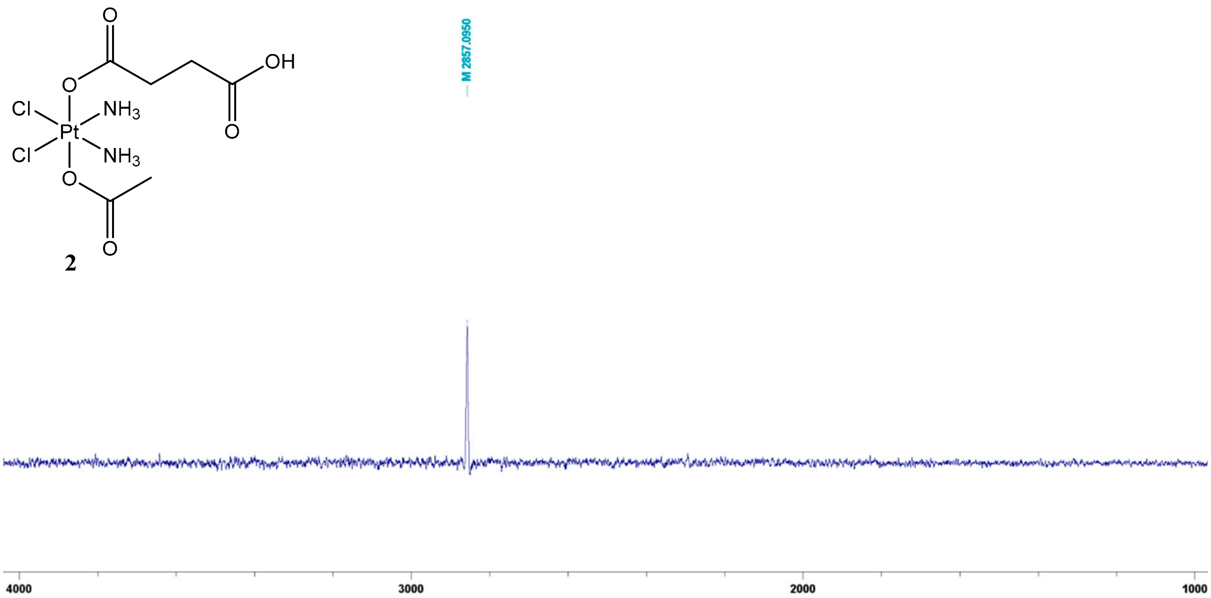

Figure S3. ^{195}Pt NMR spectrum of complex **2** in d$_6$-DMSO.

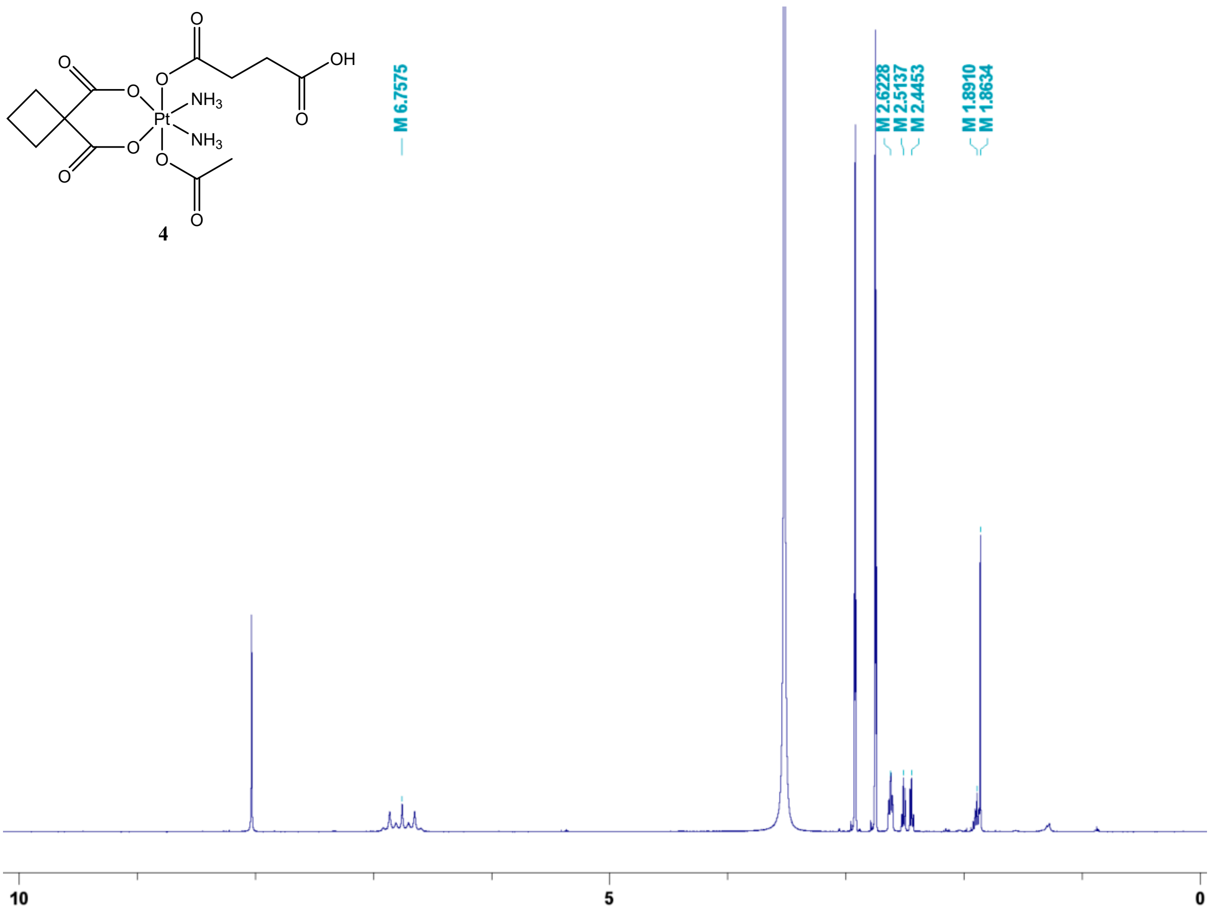

Figure S4. ^{1}H NMR spectrum of complex **4** in d_7-DMF.

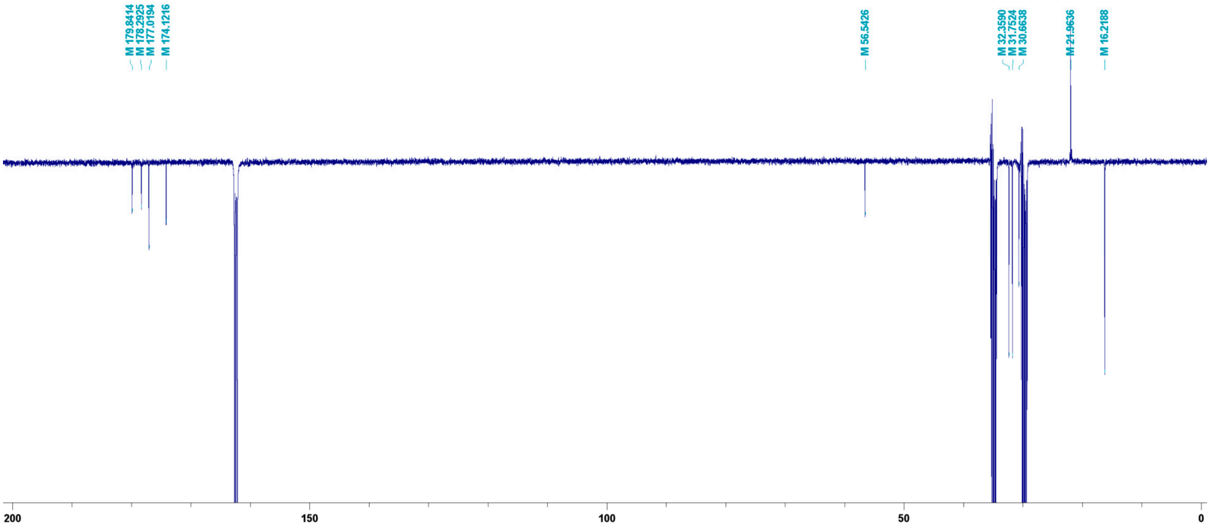

Figure S5. ^{13}C NMR spectrum of complex **4** in d_7-DMF.

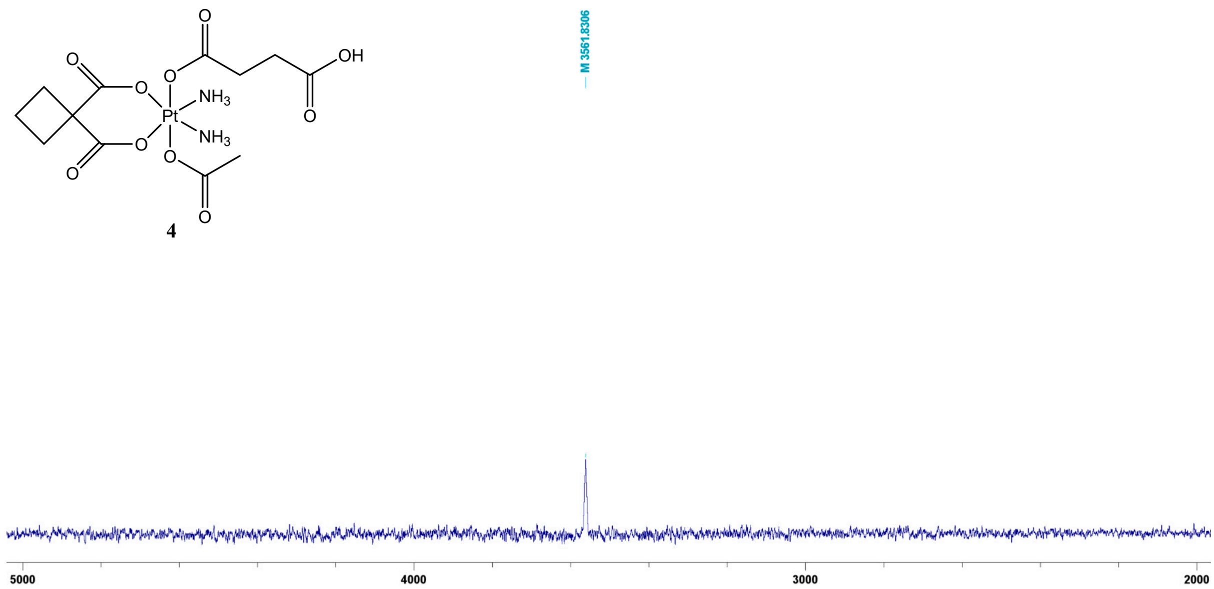

Figure S6. ^{195}Pt NMR spectrum of complex **4** in d_7-DMF.

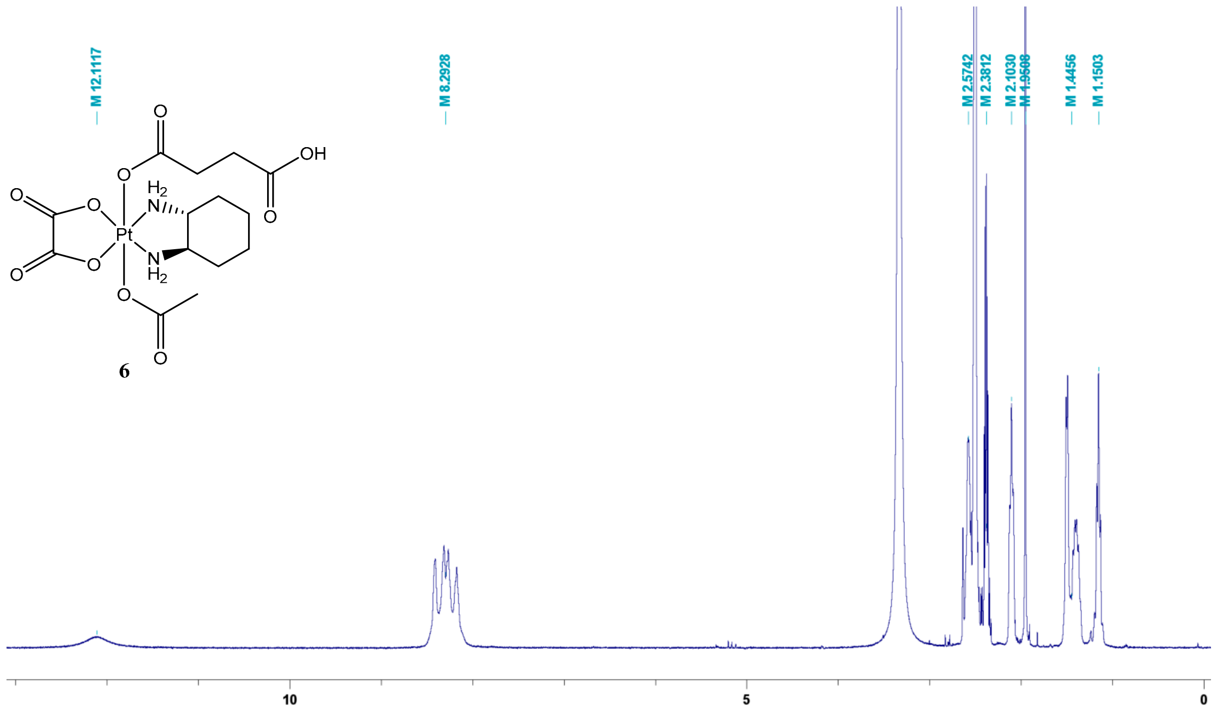

Figure S7. ^{1}H NMR spectrum of complex **6** in d_6-DMSO.

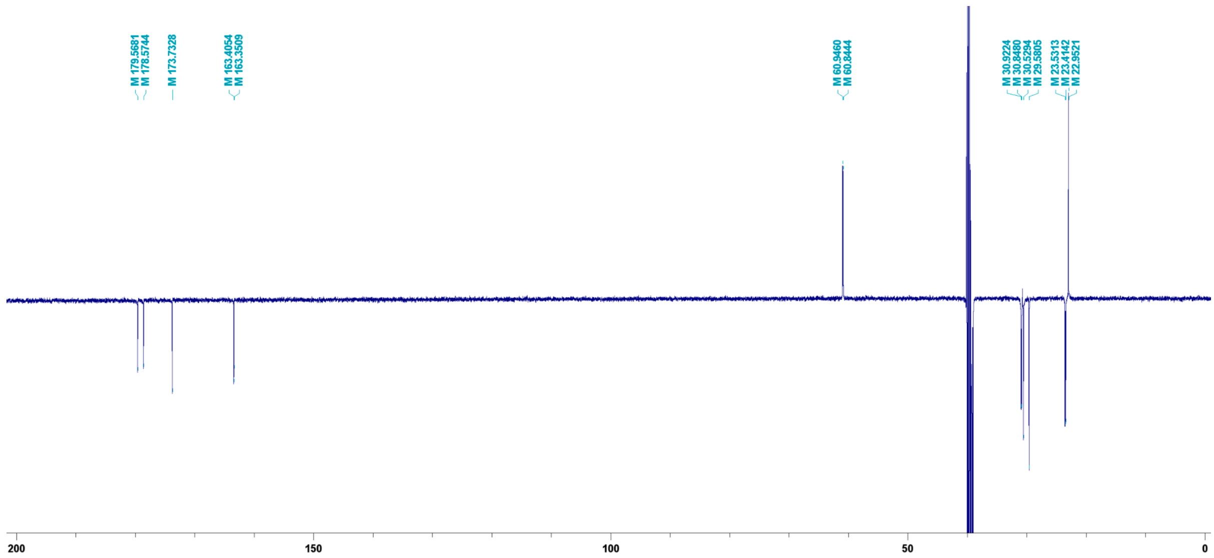

Figure S8. ^{13}C NMR spectrum of complex **6** in d_6-DMSO.

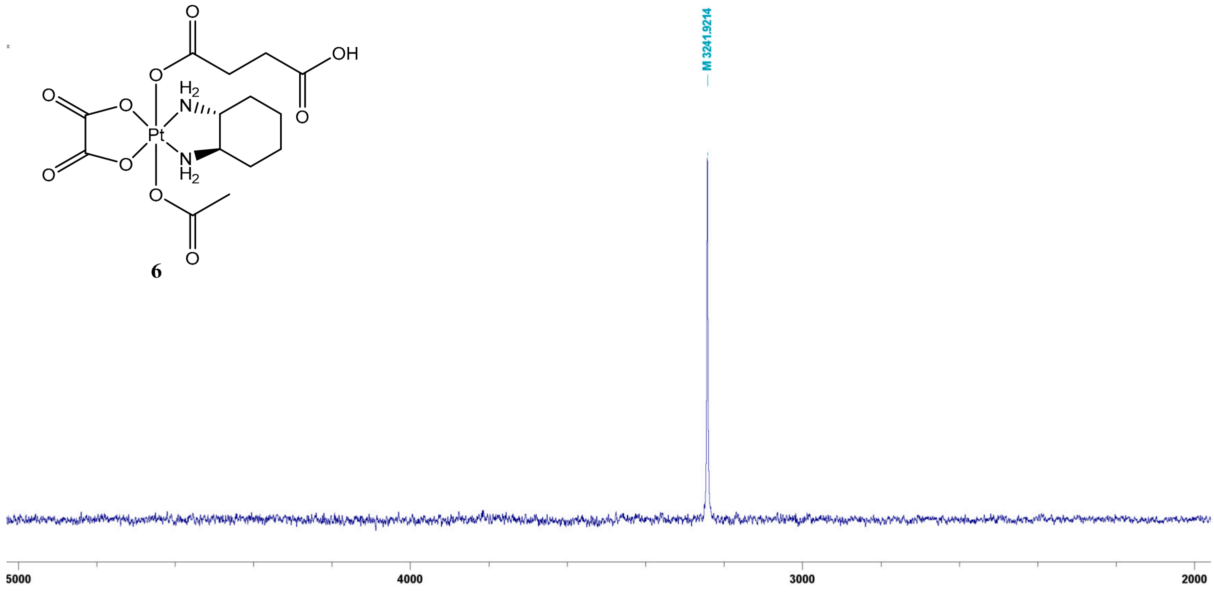

Figure S9. ^{195}Pt NMR spectrum of complex **6** in d_6-DMSO.

2. NMR spectra of dGC polymers

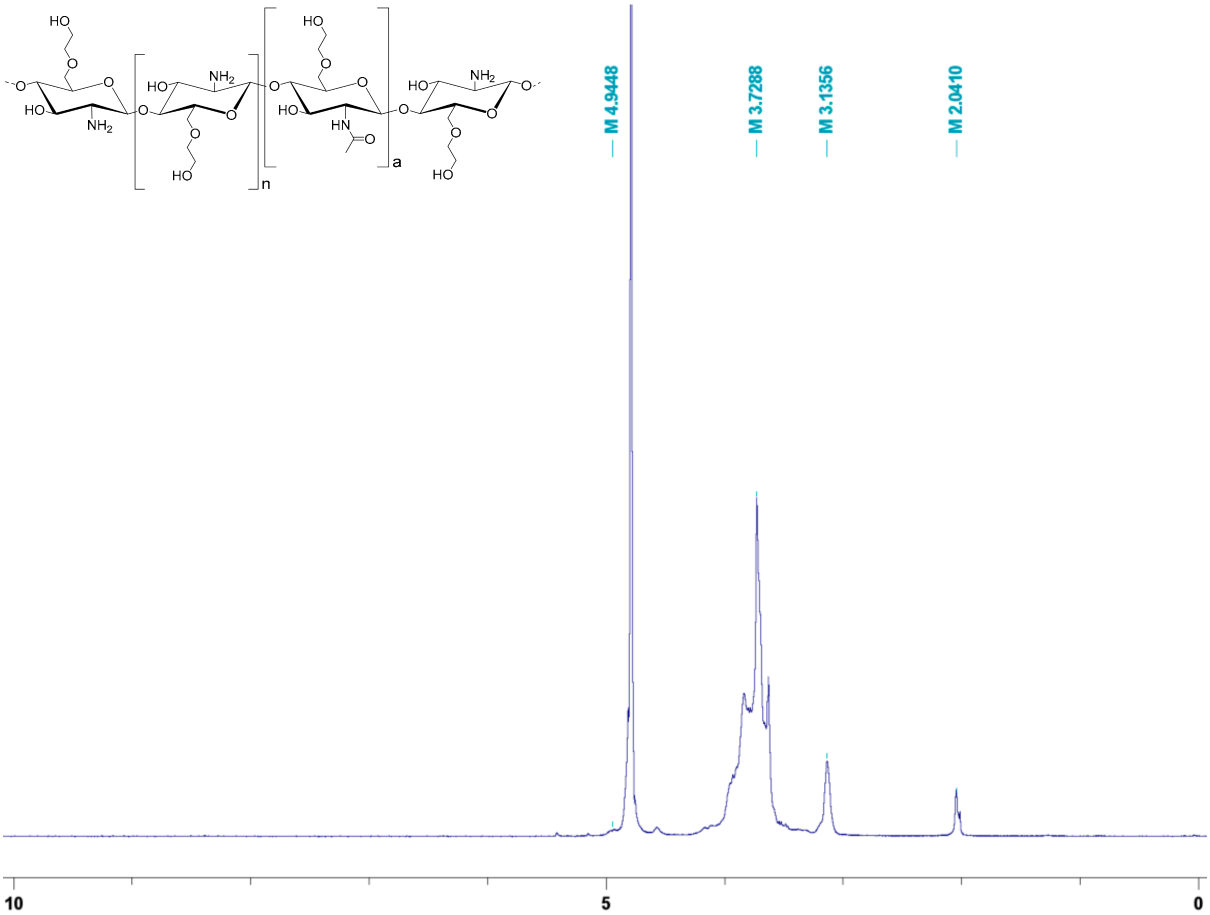

Figure S10. ^{1}H NMR spectrum of dGC polymer **P2** in D_2O.

3. NMR spectra of conjugates

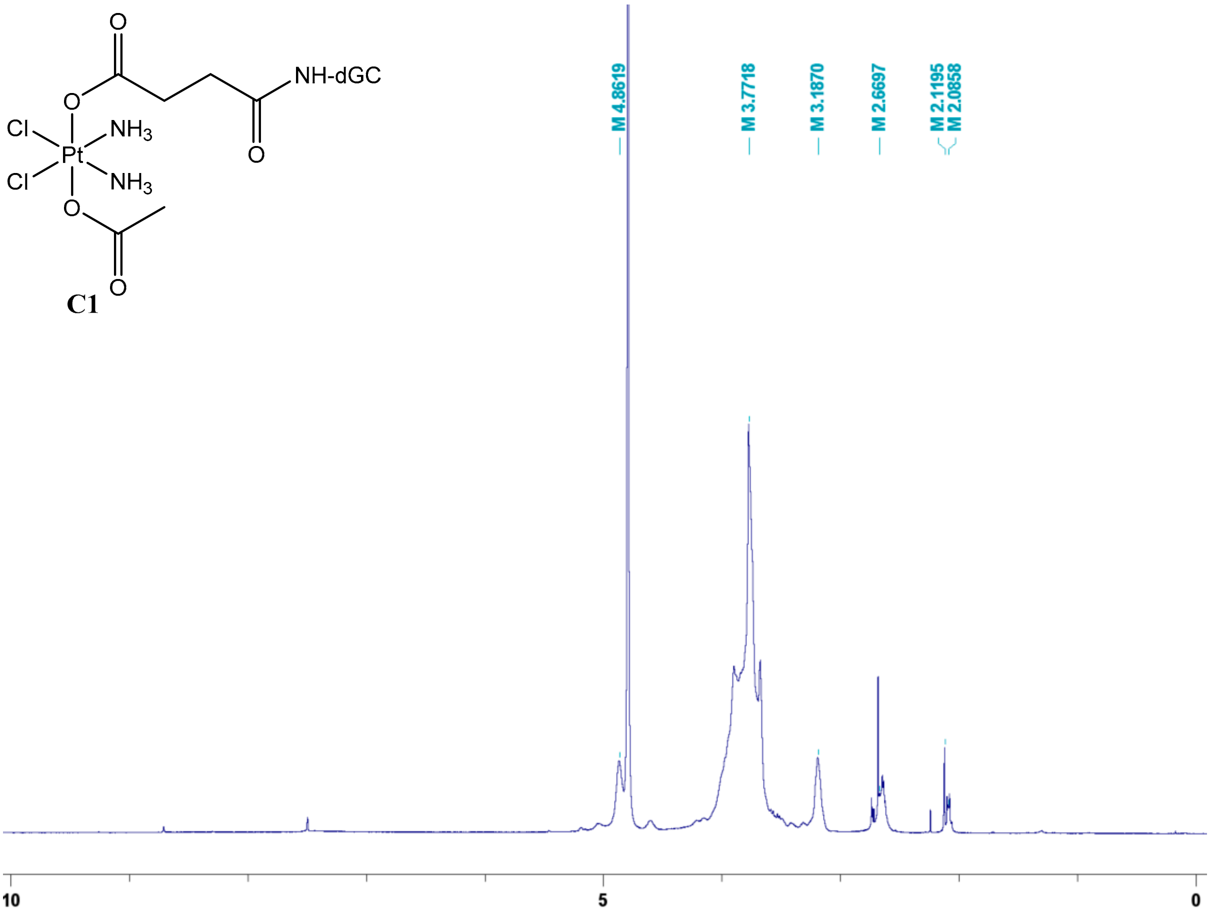

Figure S11. ^{1}H NMR spectrum of conjugate **C1** in D_2O.

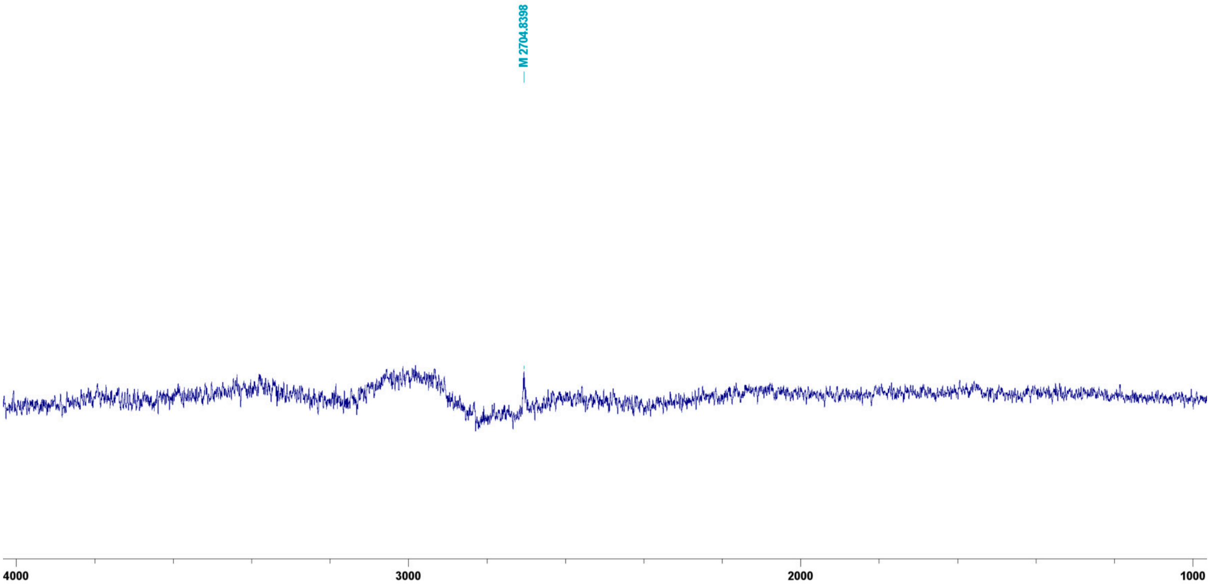

Figure S12. ^{195}Pt NMR spectrum of conjugate **C1** in D_2O.

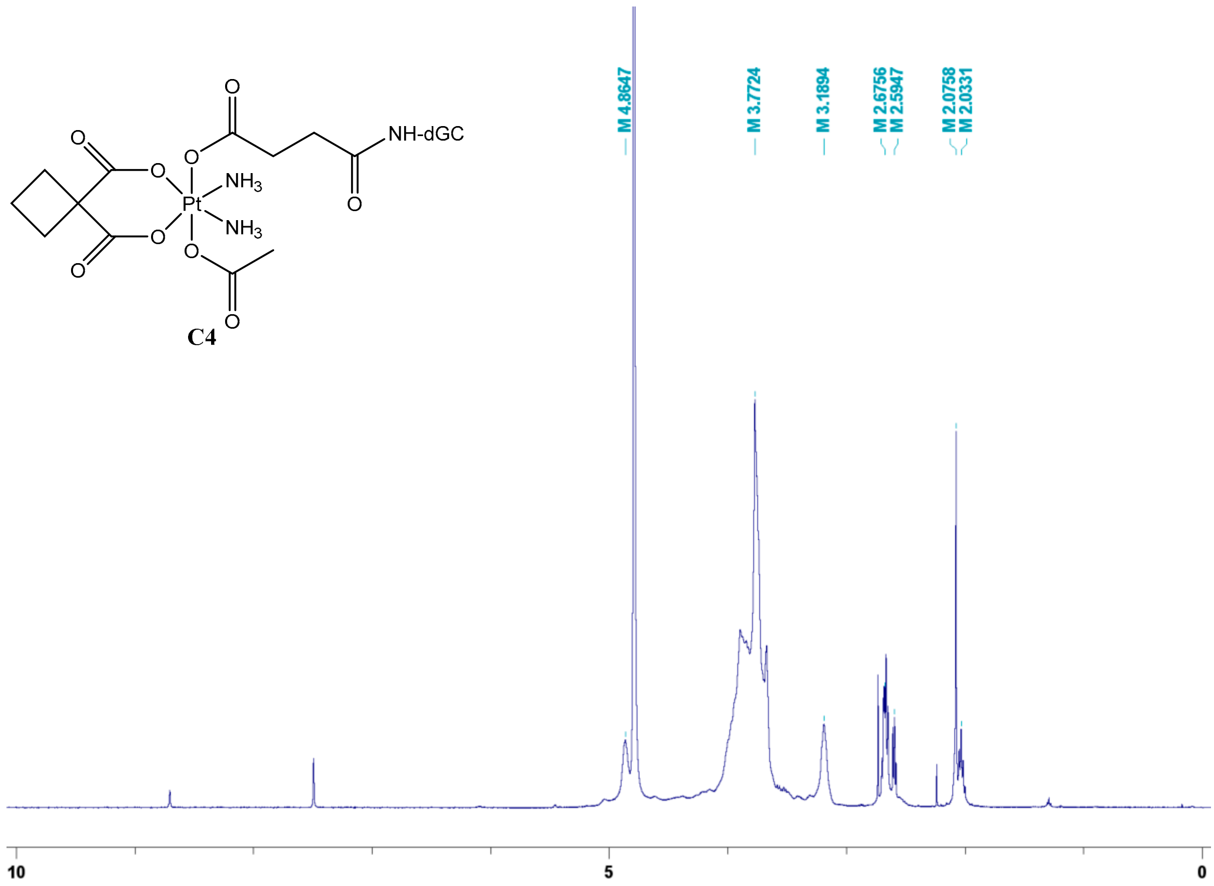

Figure S13. ^{1}H NMR spectrum of conjugate **C4** in D_2O.

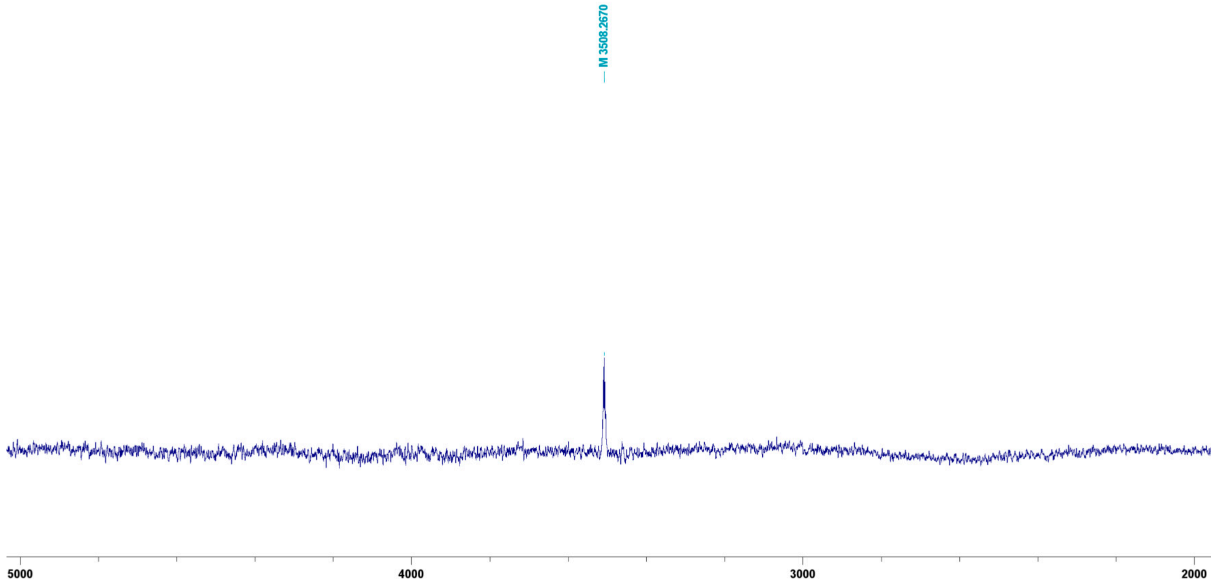

Figure S14. ^{195}Pt NMR spectrum of conjugate **C4** in D_2O.

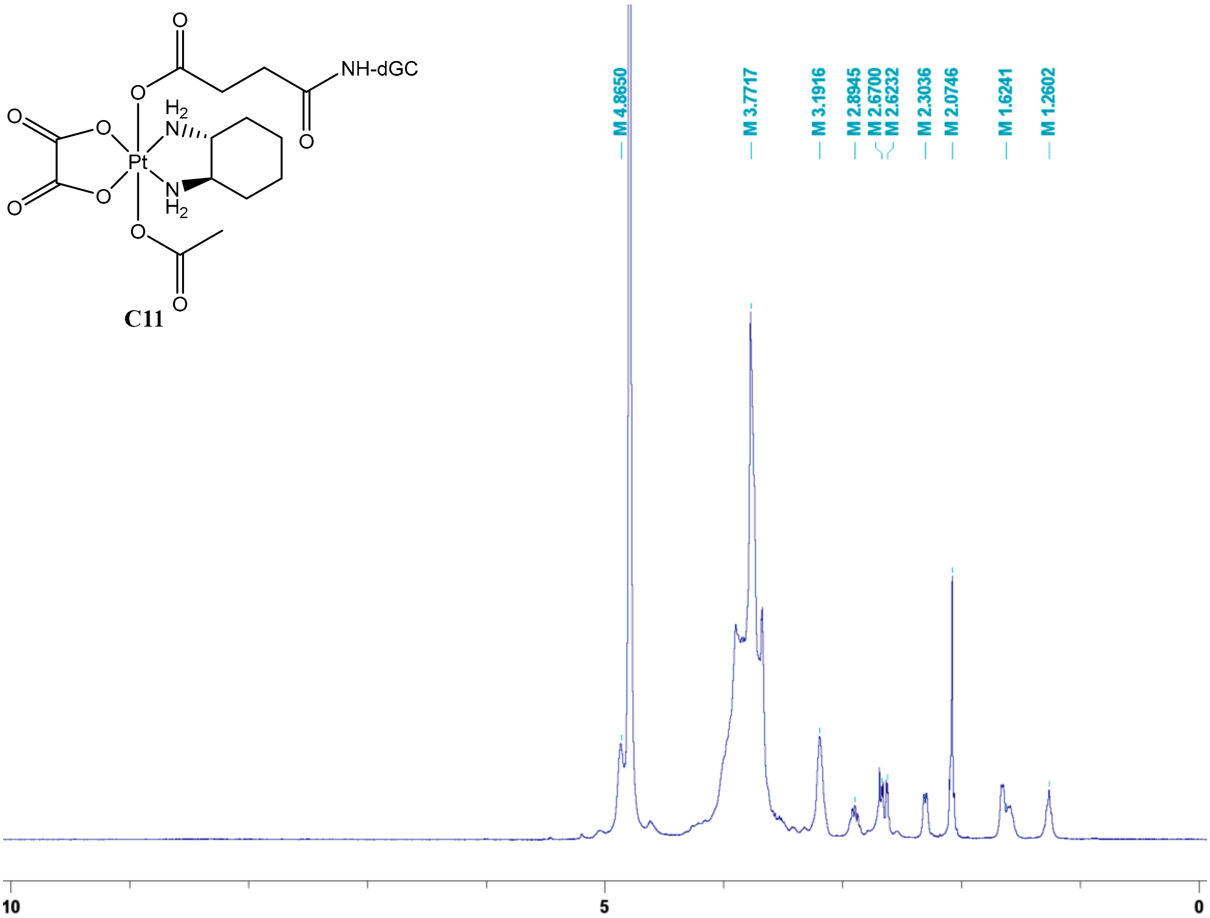

Figure S15. ^{1}H NMR spectrum of conjugate **C11** in D_2O.

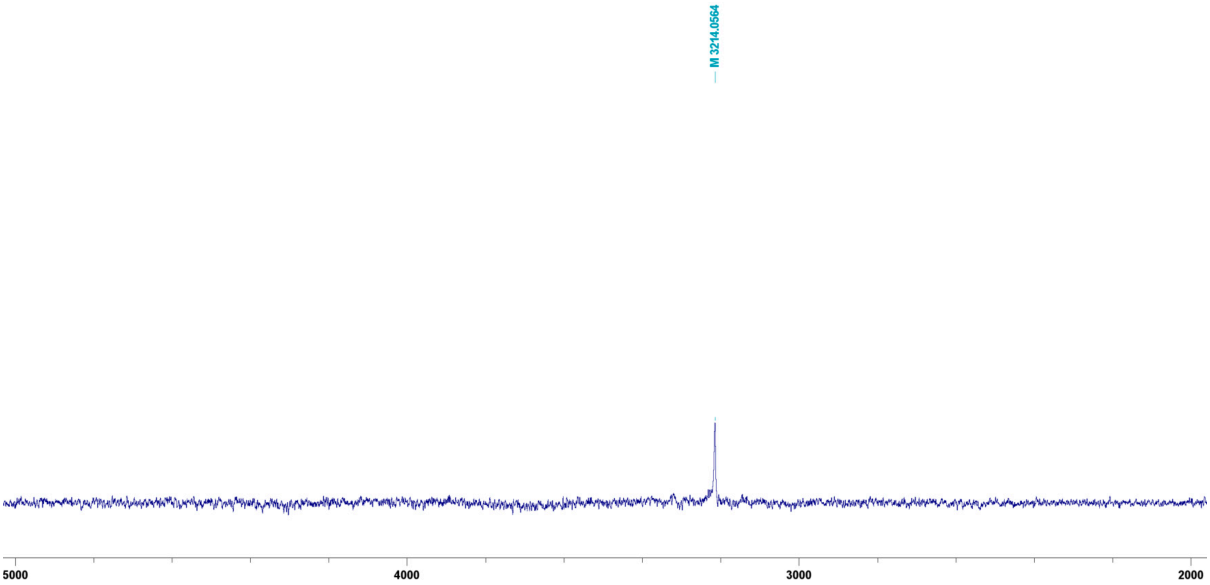

Figure S16. ^{195}Pt NMR spectrum of conjugate **C11** in D_2O.

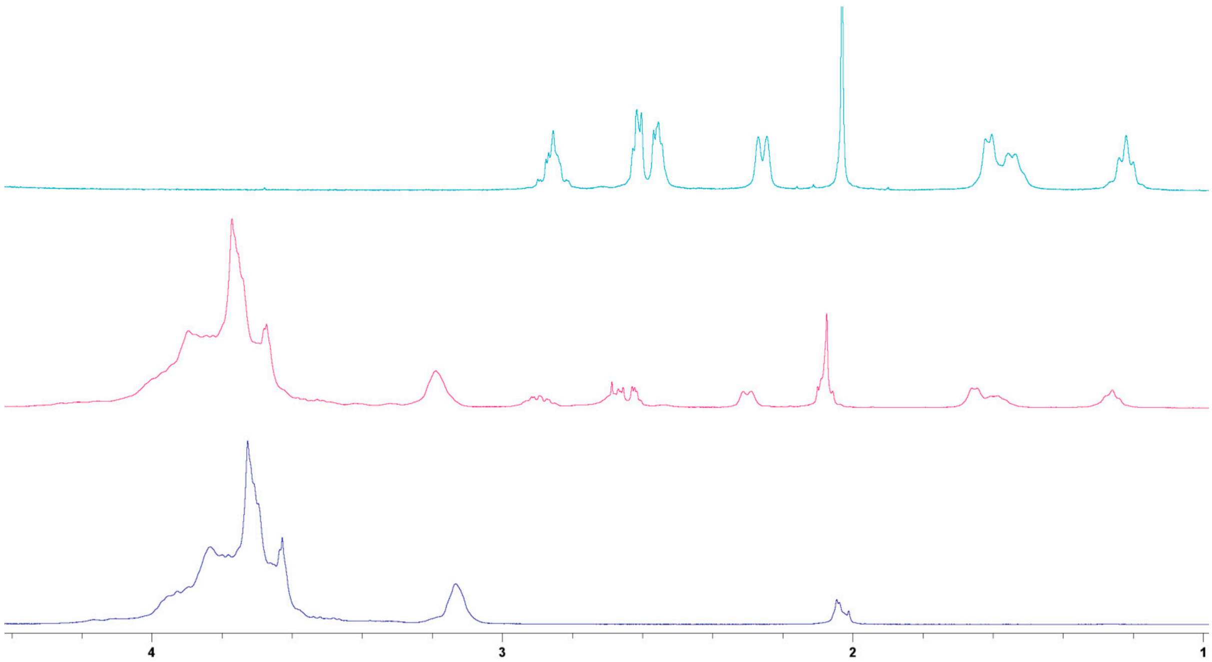

Figure S17. ^{1}H NMR spectra measured in D_2O of platinum(IV) complex **6** (above), conjugate **C11** (middle) and dGC polymer **P2** (below).

4. Concentration-effect curves

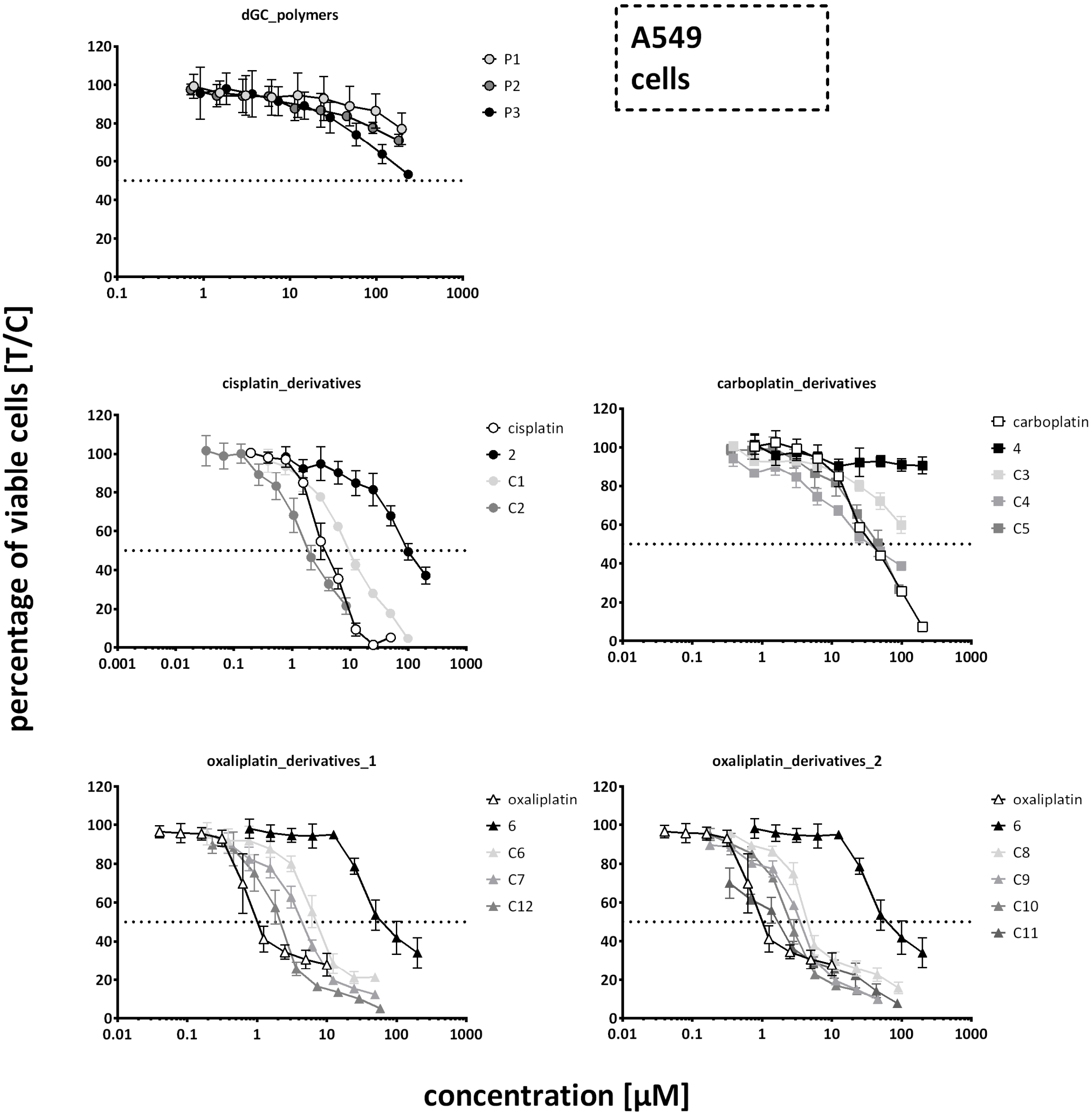

Figure S18. Concentration-effect curves in human cancer cell line A549 in MTT assays.

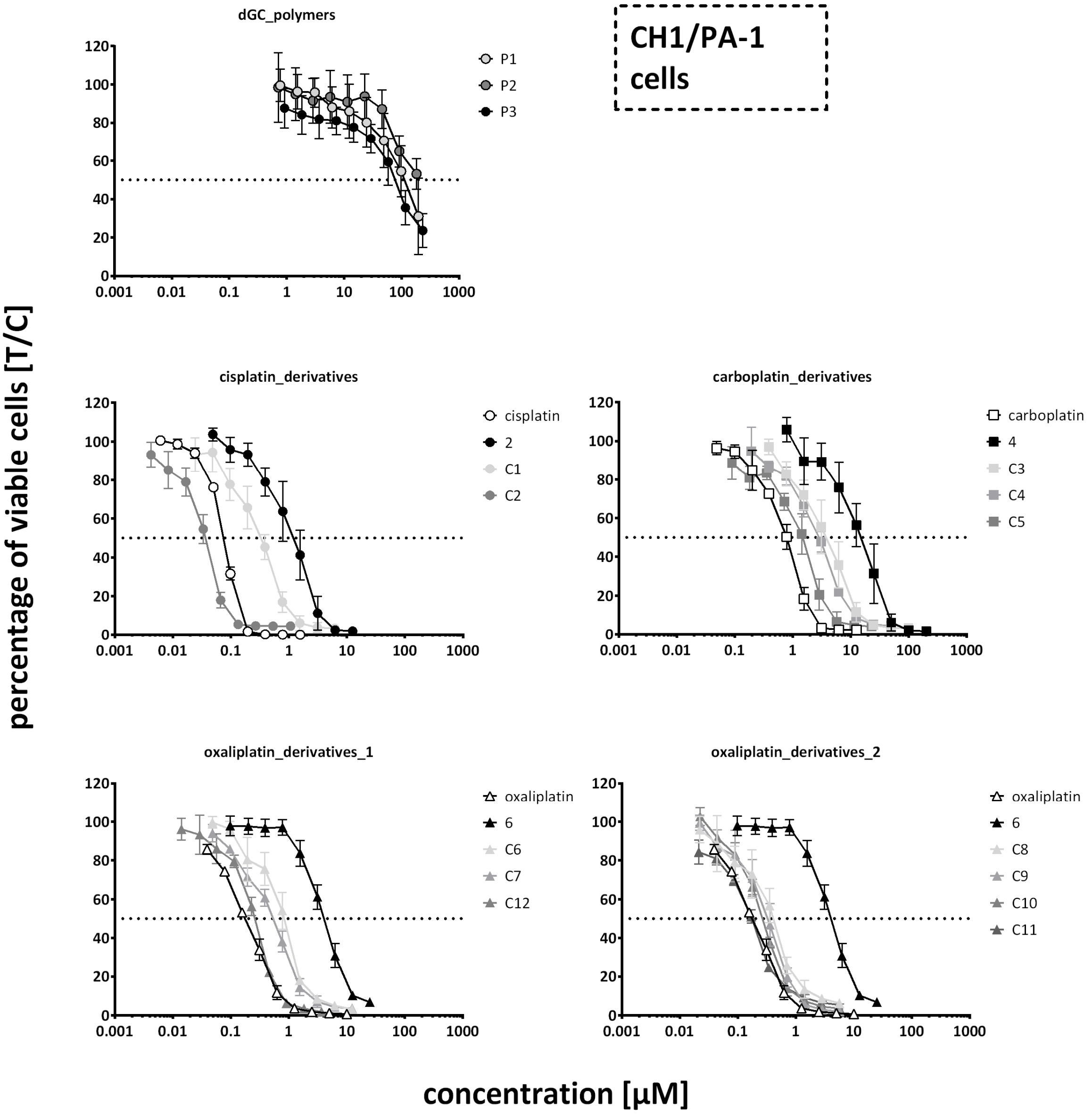

Figure S19. Concentration-effect curves in human cancer cell line CH1/PA-1 in MTT assays.

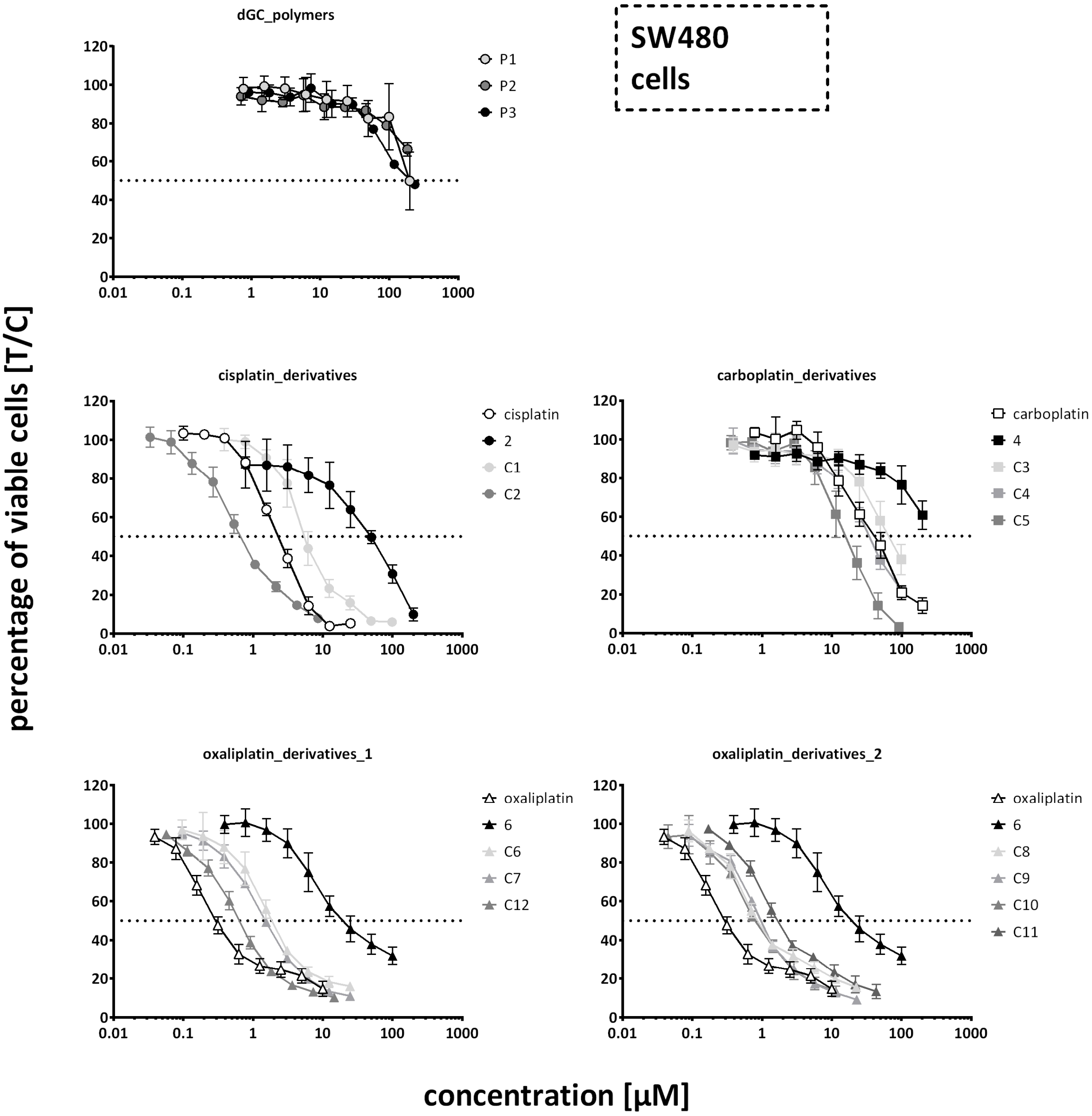

Figure S20. Concentration-effect curves in human cancer cell line SW480 in MTT assays.

5. Solubility data

Table S1. Overview of the water solubility of conjugates **C1-C12**, **S1-S5**, **V1-V3** measured by visual judgement in Milli-Q water at room temperature.

Sample	Pt(IV)	dGC	Water solubility [mg/mL]
**C1**	**2**	**P1**	>36
**C2**	**2**	**P2**	~1
**C3**	**4**	**P1**	>39
**C4**	**4**	**P1**	~14
**C5**	**4**	**P2**	~7
**C6**	**6**	**P1**	>48
**C7**	**6**	**P1**	>38
**C8**	**6**	**P2**	>19
**C9**	**6**	**P2**	>22
**C10**	**6**	**P2**	>15
**C11**	**6**	**P2**	~4
**C12**	**6**	**P3**	~2
**S1**	**2**	**P3**	<0.5
**S2**	**2**	**P3**	<0.5
**S3**	**4**	**P3**	<0.5
**S4**	**4**	**P3**	<0.5
**S5**	**6**	**P3**	<0.5
**V1**	**6**	**P1**	>55
**V2**	**6**	**P2**	>21
**V3**	**6**	**P3**	~9
